# Natural product-based therapeutic strategies against *Leishmania*: mechanistic insights and emerging host-directed approaches

**DOI:** 10.3389/fmicb.2026.1955098

**Published:** 2026-09-01

**Authors:** Pankaj Verma, Sorabh Chaudhary, Monu Monu, Tanya Aggarwal, Meenu Singh, Avinash Singh, Vivek Kumar

**Affiliations:** 1 Department of Biotechnology, School of Engineering, IILM University, Greater Noida, Uttar Pradesh, India; 2 Department of Biology, Faculty of Science and Humanities, SRM Institute of Science and Technology, Delhi-NCR Campus, Ghaziabad, Uttar Pradesh, India; 3 Department of Biotechnology, MIET, Meerut, Uttar Pradesh, India; 4 Center of Excellence in Aging and Brain Repair, USF Center for Microbiome Research, Microbiomes Institute, Morsani College of Medicine, University of South Florida, Tampa, FL, United States

**Keywords:** arginase, drug combinations, host-directed therapy, immunomodulation, leishmaniasis, macrophages, microRNAs, natural products

## Abstract

Leishmaniasis remains a major neglected tropical disease, while available treatments are constrained by toxicity, cost, prolonged administration, relapse, variable geographical efficacy, and emerging drug resistance. Natural products provide structurally diverse antileishmanial candidates capable of acting through both parasite-directed and host-mediated mechanisms. This review critically evaluates natural compounds targeting parasite membrane integrity, mitochondrial function, redox homeostasis, arginase and polyamine metabolism, sterol biosynthesis, and replication-associated enzymes. It also examines their immunomodulatory effects, including macrophage activation, nitric oxide and reactive oxygen species production, promotion of T-helper-1-associated responses, and suppression of parasite-favorable immune pathways. Particular attention is given to emerging host-directed strategies involving microRNAs that regulate autophagy, inflammatory signaling, macrophage polarization, and host lipid metabolism. Functional evidence supports selected miRNA-target axes, including miR-30a-3p-BECN1, miR-330-5p-SPTLC1, miR-1303-HMGCR, and miR-874-3p-HMGCS1. However, the therapeutic consequences of miRNA modulation are species- and disease-form-dependent, and direct evidence connecting natural-product activity with specific miRNA-mediated mechanisms remains limited. Although several natural products and natural product-drug combinations demonstrate activity against intracellular amastigotes and in animal models, much of the literature still relies on promastigote assays, crude extracts, molecular docking, or correlative immune and transcriptomic measurements. Clinical translation will require chemical standardization, direct target validation, macrophage-selective delivery, pharmacokinetic and toxicological evaluation, and demonstration of efficacy in clinically relevant infection models. Overall, natural products and host-directed interventions represent complementary therapeutic strategies, but progress will depend on moving from descriptive screening toward standardized, mechanism-based, and translationally relevant investigation.

## 1 Introduction

Leishmaniasis comprises a heterogeneous group of neglected tropical diseases caused by more than 20 human-infective species of the protozoan genus *Leishmania* and transmitted primarily through the bites of infected female phlebotomine sandflies. Its principal clinical manifestations are cutaneous leishmaniasis, which produces localized or disseminated skin lesions; mucosal or mucocutaneous leishmaniasis, which can progressively damage the mucous membranes; and visceral leishmaniasis, or kala-azar, which affects internal organs and is usually fatal when untreated. The World Health Organization estimates that approximately 600,000–1 million new cutaneous cases and 50,000–90,000 new visceral cases occur annually, although substantial underreporting limits accurate assessment of the global burden (Clem, 2010). The disease disproportionately affects populations experiencing poverty, malnutrition, population displacement, inadequate housing and limited access to healthcare. Nutritional impairment can also compromise host immunity and facilitate early parasite visceralization, further connecting disease severity with socioeconomic vulnerability (Ibrahim et al., 2013).

Following transmission, *Leishmania* parasites establish infection principally within macrophages, where they remodel cellular signaling, metabolism, antimicrobial activity and immune responses to support intracellular persistence (Gurjar et al., 2025). Disease outcome therefore depends not only on parasite species and virulence but also on the balance between protective and permissive host responses. This biological dependence on the host distinguishes leishmaniasis from infections in which pathogen elimination is achieved primarily through direct antimicrobial activity and provides a rationale for combining parasite-directed treatment with host-directed therapeutic approaches.

The available antileishmanial drugs—including pentavalent antimonials, amphotericin B formulations, miltefosine, paromomycin and pentamidine—remain limited by toxicity, geographical variation in efficacy, parenteral administration, prolonged treatment, cost, relapse and emerging or established resistance (Ashutosh and Goyal, 2007; Sundar and Chakravarty, 2010, 2015; Nagle et al., 2014; Majoor et al., 2025; Verma et al., 2025). Liposomal amphotericin B has improved the efficacy and tolerability of visceral leishmaniasis treatment, but its cost, intravenous administration and formulation requirements restrict access in many endemic settings (Sundar and Chakravarty, 2010). Miltefosine provides an oral alternative, but treatment failure, relapse, gastrointestinal toxicity, teratogenicity and the potential selection of resistant parasites remain concerns. These limitations underscore the need for chemically and mechanistically distinct therapeutic candidates that are effective against intracellular amastigotes and suitable for use in resource-limited settings.

Natural products constitute an important source of structurally diverse bioactive molecules and have yielded numerous compounds with antileishmanial activity (Gervazoni et al., 2020; Carter et al., 2021). Reported mechanisms include disruption of parasite membranes and mitochondrial function, induction of oxidative stress, interference with DNA replication and signaling, and inhibition of parasite enzymes involved in redox balance, arginine metabolism and polyamine biosynthesis. Certain natural compounds also influence macrophage activation, cytokine production, nitric oxide generation and other host responses associated with intracellular parasite control. Formononetin, for example, has demonstrated both direct activity against *L. tropica* and increased NO, IFN-γ and iNOS-associated responses in infected macrophages (Mahmoudvand et al., 2023). However, the evidence supporting many other candidates remains limited to promastigote assays, crude extracts, molecular docking or expression-level associations, and these findings do not establish intracellular efficacy or direct target engagement.

Host-directed therapy has further broadened the conceptual framework for natural product-based intervention. *Leishmania* modifies macrophage signaling, autophagy, lipid metabolism, cytokine responses and microRNA expression to maintain intracellular infection (Singh et al., 2016; Acuña et al., 2020; Gurjar et al., 2025). Modulating these host pathways could complement direct parasite killing and potentially improve treatment efficacy. Nevertheless, host-directed intervention also presents risks because immune and metabolic pathways exploited by the parasite perform essential physiological functions. Moreover, evidence that natural products exert antileishmanial activity through specific host miRNAs remains sparse and is largely extrapolated from cancer or non-infectious inflammatory models.

This review critically evaluates natural product-based therapeutic strategies against *Leishmania*, with emphasis on the strength of experimental evidence supporting their mechanisms of action. It examines natural compounds targeting parasite-specific pathways, their immunomodulatory and dual host–parasite effects, emerging miRNA-directed strategies and the potential value of combination or formulation-assisted therapies. Particular attention is given to distinguishing experimentally validated mechanisms from molecular docking, computational prediction and correlative immune or miRNA responses. By integrating parasite-directed and host-directed evidence, the review identifies the principal mechanistic, pharmacological and translational gaps that must be addressed before natural product-derived candidates can progress toward clinical application.

In this review, “natural products” is used as an overarching term encompassing crude extracts, standardized fractions, and purified substances obtained from natural sources. “Natural compounds” refers to chemically defined constituents, whereas “phytochemicals” specifically denotes plant-derived compounds. “Host-directed therapy” refers to interventions that modulate host pathways to promote parasite control; where pathway dependence has not been experimentally demonstrated, the more cautious terms “host-modulatory” or “immunomodulatory effects” are used.

## 2 Literature search strategy

This critical narrative review was developed through searches of PubMed, Scopus, Web of Science and Google Scholar up to July 2026. Search terms included “*Leishmania*,” “natural products,” “antileishmanial activity,” “intracellular amastigotes,” “immunomodulation,” “host-directed therapy,” “microRNA,” “arginase,” and “combination therapy.” Priority was given to peer-reviewed primary studies using infected macrophages, intracellular amastigotes or animal models. Promastigote-only and computational studies were included as preliminary evidence but distinguished from experimentally validated mechanisms. Relevant reviews and reference lists were additionally screened to identify eligible studies. This was a critical narrative synthesis; therefore, no formal meta-analysis was performed.

## 3 Biology of *Leishmania* infection and host–parasite interaction

### 3.1 Life cycle of *Leishmania*

*Leishmania* parasites are transmitted to mammalian hosts through the bite of infected female phlebotomine sandflies. During a blood meal, the sandfly inoculates metacyclic promastigotes into the dermis. These elongated, flagellated forms are subsequently taken up by macrophages and other mononuclear phagocytic cells. Within the acidic phagolysosomal compartment, promastigotes differentiate into small, non-flagellated amastigotes, which constitute the principal replicative stage in the mammalian host. Amastigotes multiply intracellularly and can spread to neighboring phagocytic cells, with the affected tissues and clinical manifestations depending on the infecting *Leishmania* species and host immune response (Gossage et al., 2003; Rogers and Bates, 2007; Wilson et al., 2010).

The parasite cycle continues when an uninfected sandfly takes a blood meal from an infected mammalian host and ingests parasite-containing cells. Within the sandfly midgut, amastigotes transform into promastigotes, undergo successive developmental stages and migrate toward the anterior gut and proboscis. Differentiation into infective metacyclic promastigotes completes the vector phase and enables transmission during a subsequent blood meal (Gossage et al., 2003; Rogers and Bates, 2007; Kaye and Scott, 2011). Thus, the parasite alternates between an extracellular, flagellated promastigote stage in the sandfly and an intracellular amastigote stage in the mammalian host.

### 3.2 Macrophage infection and intracellular survival

Following inoculation into the dermis, *Leishmania* promastigotes encounter neutrophils, macrophages, dendritic cells and other resident immune cells. Macrophages recognize and internalize the parasites through several surface receptors, including complement receptors, mannose receptors, fibronectin receptors and selected Toll-like receptors. However, parasite uptake does not necessarily result in elimination. Instead, *Leishmania* exploits the phagocytic process to enter a host cell that supports its differentiation and intracellular persistence (De Veer et al., 2003; Tuon et al., 2008; Liu and Uzonna, 2012; Von Stebut and Tenzer, 2018).

Within macrophages, promastigotes are enclosed in phagosomal compartments and subsequently differentiate into amastigotes that are adapted to the acidic and nutrient-restricted phagolysosomal environment. Parasite surface and secreted molecules, particularly lipophosphoglycan and the metalloprotease GP63, modify host-cell signaling and phagosome properties. Lipophosphoglycan can delay phagosome maturation, alter membrane organization and interfere with NADPH oxidase assembly, thereby limiting the production of parasite-killing reactive oxygen species. GP63 can cleave or modify host signaling proteins and contribute to impaired inflammatory and antimicrobial responses (Moradin and Descoteaux, 2012; Olivier et al., 2012; Sindhu et al., 2018; Verma et al., 2019).

The ability of *Leishmania* to persist within macrophages varies according to parasite species, developmental stage and host-cell phenotype. Metacyclic promastigotes can alter phagosomal composition during the early phase of infection, whereas intracellular amastigotes employ additional metabolic and immune-evasion mechanisms to maintain chronic infection (Moradin and Descoteaux, 2012; Matte et al., 2021). Consequently, therapies that show activity against extracellular promastigotes may fail if they cannot enter macrophages, accumulate within phagolysosomes or remain active under acidic intracellular conditions. The macrophage is therefore both the principal cellular niche of *Leishmania* and an important target for parasite-directed drug delivery and host-directed immunomodulation.

### 3.3 Host immune response to *Leishmania*

The outcome of *Leishmania* infection is determined by interactions among macrophages, dendritic cells, neutrophils, natural killer cells and antigen-specific T cells. Following parasite recognition and uptake, dendritic cells and macrophages produce cytokines that influence the development of protective or parasite-permissive immune responses. IL-12 promotes IFN-γ production by natural killer and T cells, leading to macrophage activation and induction of antimicrobial mechanisms, particularly inducible nitric oxide synthase, nitric oxide and reactive oxygen species. These responses contribute to intracellular parasite control, although their relative importance varies among *Leishmania* species, host tissues and experimental models (Alexander and Brombacher, 2012; Liu and Uzonna, 2012; Kima and Soong, 2013; Olekhnovitch and Bousso, 2015).

Conversely, IL-10 and other immunoregulatory pathways can suppress macrophage activation and permit chronic parasite persistence. IL-10 is particularly important in human visceral leishmaniasis, where it limits inflammatory cytokine production and macrophage-mediated parasite killing (Nylén and Sacks, 2007). Increased arginase activity may further favor parasite survival by competing with inducible nitric oxide synthase for L-arginine and directing the substrate toward ornithine and polyamine synthesis. These metabolites support parasite proliferation and redox homeostasis (Iniesta et al., 2005; Pessenda and Da Silva, 2020).

The traditional Th1/Th2 model provides a useful framework for understanding experimental *L. major* infection, in which Th1-associated responses are generally protective and Th2-associated responses can increase susceptibility. However, this binary model does not fully explain human leishmaniasis. The functions of IL-4, IL-13, IL-10 and IFN-γ vary according to parasite species, clinical manifestation, infection stage and tissue microenvironment (Alexander and Brombacher, 2012; Hurdayal and Brombacher, 2014; Bogdan, 2020). For example, strong IFN-γ-associated responses may support parasite control, but excessive or poorly regulated inflammatory activity can contribute to tissue injury in mucosal and some forms of cutaneous leishmaniasis (Bacellar et al., 2002; Scott and Novais, 2016; Tiwari et al., 2016).

**Figure 1:**
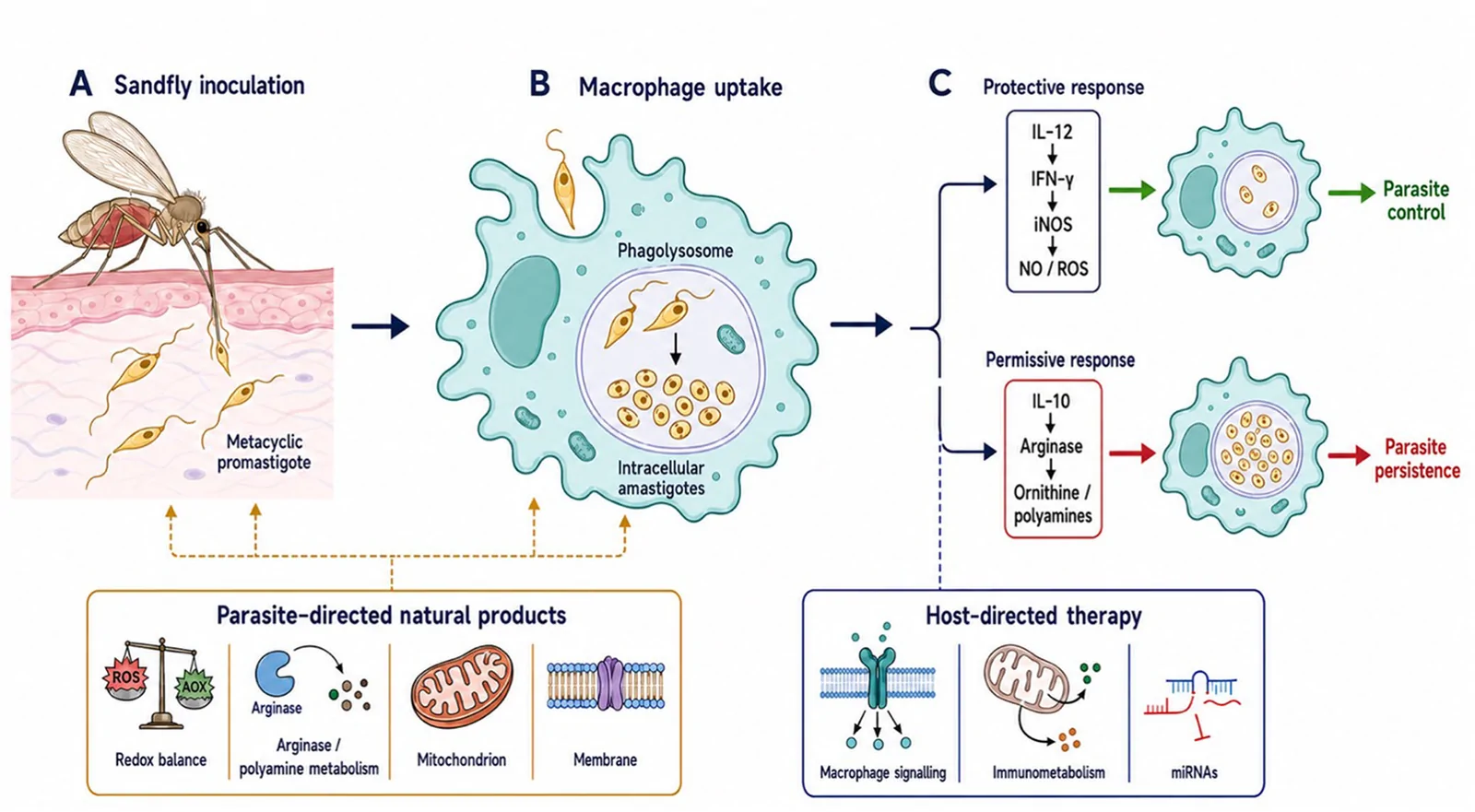
Life cycle, intracellular survival and therapeutically relevant host–parasite interactions during *Leishmania* infection. **(A)** Infected female phlebotomine sandflies inoculate metacyclic promastigotes into the dermis. **(B)** Promastigotes are internalized by macrophages and differentiate into replicating amastigotes within phagolysosomal compartments. Natural products may directly interfere with parasite redox homeostasis, arginase–polyamine metabolism, mitochondrial function and membrane integrity. **(C)** IL-12–IFN-γ-iNOS signaling promotes nitric oxide and reactive oxygen species production and contributes to parasite control, whereas IL-10 and arginase-associated ornithine and polyamine metabolism favor parasite persistence. Host-directed approaches may modulate macrophage signaling, immunometabolism and miRNA-regulated pathways. AOX, antioxidant defense; IFN-γ, interferon-γ; IL, interleukin; iNOS, inducible nitric oxide synthase; NO, nitric oxide; ROS, reactive oxygen species.

Therefore, immune activation should not automatically be interpreted as a beneficial therapeutic outcome. Natural products proposed as immunomodulatory agents must demonstrate that changes in cytokines, macrophage polarization, nitric oxide or reactive oxygen species are accompanied by reduced intracellular parasite burden without excessive host-cell toxicity or inflammatory tissue damage. This distinction is particularly important when evaluating host-directed therapies, because the same immune pathway may contribute to protection in one form of leishmaniasis but to pathology in another. Collectively, the parasite life cycle, intracellular adaptation and balance between protective and parasite-permissive host responses provide the biological framework for parasite-directed and host-directed natural product-based interventions (Figure 1).

## 4 *In vitro* and *in vivo* efficacy of natural products

Natural products represent an important source of structurally diverse bioactive compounds for antileishmanial drug discovery. Their chemical diversity enables interactions with multiple parasite and host pathways, including redox homeostasis, mitochondrial function, polyamine metabolism, membrane integrity and immune signaling. Interest in natural products has increased because existing antileishmanial drugs are limited by toxicity, parenteral administration, variable geographical efficacy, relapse and drug resistance (Tullius Scotti et al., 2016; Gervazoni et al., 2020; Elawad et al., 2023).

However, natural origin should not be considered evidence of safety or therapeutic effectiveness. The majority of reported natural compounds have been evaluated only through computational analyses, enzyme-inhibition experiments or *in vitro* parasite assays. Relatively few have progressed to animal evaluation, pharmacokinetic characterization or clinical investigation. Natural products should therefore be considered sources of potential therapeutic leads rather than established alternatives to conventional antileishmanial treatment.

### 4.1 *In vitro* and *in vivo* evidences for the antileishmanial activity of natural products

Medicinal plants are the most extensively investigated sources of natural antileishmanial compounds. Plant-derived alkaloids, flavonoids, terpenoids, quinones, sterols, lignans, saponins and other phenolic metabolites have demonstrated activity against different *Leishmania* species (Yamamoto et al., 2015; Tullius Scotti et al., 2016; Gervazoni et al., 2020; Elawad et al., 2023). A review of studies published between 2015 and 2021 identified 94 medicinal plant species with reported antileishmanial activity; however, approximately 84% of the included investigations were conducted *in vitro*, and only a subset of tested extracts demonstrated favorable host-cell selectivity (Hassan et al., 2022). This imbalance illustrates the considerable gap between initial phytochemical screening and translational development.

Several purified or chemically defined plant-derived compounds have demonstrated experimentally supported mechanisms. Quercetin induces reactive oxygen species production and mitochondrial dysfunction in *L*. amazonensis (Fonseca-Silva et al., 2011). Fisetin, luteolin and structurally related flavonoids inhibit arginase from *L. amazonensis*, although enzyme inhibition alone does not establish intracellular or efficacy *in vivo* (Manjolin et al., 2013). Ursolic acid has shown activity against *L. amazonensis in vitro* and reduced disease progression in an experimental model of cutaneous leishmaniasis (Yamamoto et al., 2015). Similarly, β-sitosterol derived from *Corchorus capsularis* was reported to target trypanothione reductase and exert antileishmanial activity against *L. donovani* (Pramanik et al., 2020). These examples provide stronger evidence than crude-extract screening because the active constituents and proposed mechanisms were investigated experimentally.

Microorganisms are another important source of antiparasitic metabolites. Amphotericin B, a polyene macrolide originally derived from *Streptomyces nodosus*, demonstrates that microbial natural products can be successfully developed into clinically effective antileishmanial agents. Nevertheless, most recently identified metabolites from bacteria, actinomycetes, endophytic fungi and other microorganisms remain at an early discovery stage, with limited information concerning intracellular activity, selectivity, pharmacokinetics and efficacy *in vivo*.

Marine organisms, including algae, sponges, tunicates and marine-associated microorganisms, provide additional chemically distinctive metabolites, such as alkaloids, terpenoids, peptides and sulphated polysaccharides (Mayer et al., 2024; Cheng et al., 2025). Some marine-derived compounds have demonstrated antiprotozoal activity through membrane disruption, oxidative damage or inhibition of essential metabolic pathways. However, evidence against clinically relevant intracellular *Leishmania* amastigotes remains comparatively limited. Sustainable sourcing, structural complexity, compound availability and formulation may also restrict their subsequent development.

Thus, the diversity of natural sources provides a broad platform for antileishmanial discovery, but the quality of evidence varies considerably. Purified compounds with demonstrated intracellular activity, acceptable host-cell selectivity and reproducible efficacy *in vivo* should be prioritized over crude extracts or candidates supported only by computational predictions.

### 4.2 *In vitro* and *in vivo* efficacy of natural products

*In vitro* assays provide an essential first step for identifying natural products with antileishmanial activity. Extracellular promastigotes are frequently used because they can be cultivated and screened relatively easily. However, intracellular amastigotes residing within macrophages represent the clinically relevant mammalian stage and provide a more meaningful model for evaluating therapeutic potential. A compound that is active against promastigotes may be ineffective against amastigotes because of differences in parasite metabolism, drug susceptibility and intracellular accessibility. Therefore, promastigote assays should be regarded as preliminary screening rather than evidence of therapeutic efficacy (Scott and Novais, 2016; Elawad et al., 2023).

Several chemically defined natural compounds have demonstrated activity beyond computational prediction. Quercetin induces reactive oxygen species production, mitochondrial dysfunction and parasite death in *L. amazonensis* (Fonseca-Silva et al., 2011). Holanamine, a steroidal alkaloid isolated from *Holarrhena pubescens*, inhibits *L. donovani* growth by targeting DNA topoisomerase IB (Goel et al., 2023). β-Sitosterol derived from *Corchorus capsularis* has demonstrated activity against *L. donovani* through inhibition of trypanothione reductase (Pramanik et al., 2020). Ursolic acid has shown activity against *L. amazonensis* promastigotes and intracellular amastigotes and reduced lesion development and parasite burden in an experimental model of cutaneous leishmaniasis (Yamamoto et al., 2015). These studies provide stronger evidence than docking or enzyme-inhibition analyses because they demonstrate cellular or *in vivo* activity. Nevertheless, differences in parasite species, host cells, assay conditions and compound exposure limit direct comparison among studies.

Animal models provide important information regarding efficacy, toxicity, tissue distribution and immune-mediated effects. In a direct comparison, berberine-loaded liposomes increased the selectivity index by reducing macrophage cytotoxicity, enhanced berberine accumulation in the liver and spleen, and produced greater reductions in tissue parasite burden than free berberine in *L. infantum*-infected BALB/c mice (Calvo et al., 2020). However, these findings are specific to the tested formulation and treatment conditions and do not establish complete parasite elimination, sustained post-treatment efficacy, or long-term safety (Calvo et al., 2020). Ursolic acid also demonstrated therapeutic effects in experimental cutaneous leishmaniasis (Yamamoto et al., 2015). However, activity in rodents does not guarantee clinical effectiveness because disease progression, immune responses and drug disposition differ between experimental animals and humans. Moreover, many studies report reduced lesion size or organ parasite burden without comprehensive pharmacokinetic, dose-ranging or long-term toxicity analyses.

Natural compounds may also influence host responses. Curcumin increased IFN-γ, TNF-α and inducible nitric oxide synthase expression in peripheral blood mononuclear cells infected with *L. major* (Alinejad et al., 2022). However, altered cytokine or enzyme expression alone does not demonstrate parasite clearance, and immunomodulatory findings should be accompanied by measurements of intracellular parasite burden and host-cell toxicity. Similarly, improved activity following nanoparticle or liposomal delivery must be interpreted alongside carrier toxicity, drug-release behavior, biodistribution and formulation stability (Calvo et al., 2020; Dourado et al., 2024).

Overall, the evidence remains predominantly preclinical. A recent synthesis of natural antileishmanial studies reported that most candidates were examined only *in vitro*, relatively few progressed to animal evaluation, and only a subset demonstrated favorable selectivity against host cells. Consequently, the most promising candidates are those supported by reproducible intracellular-amastigote activity, an acceptable selectivity index, defined molecular targets and efficacy *in vivo* at pharmacologically achievable doses. Clinical efficacy should not be claimed until these requirements are followed by appropriate pharmacokinetic, toxicological and controlled human studies. Some studies are summarized in Table 1.

**Table 1:** Evidence supporting representative natural product-based interventions against *Leishmania*.

Natural product/ compound or formulation	Source or chemical class	Leishmania species	Experimental model	Reported mechanism or effect	Evidence level	Major limitation	Ref.
Quercetin	Flavonoid	*L. amazonensis*	Promastigotes and intracellular amastigotes	ROS accumulation, mitochondrial dysfunction, and parasite death in promastigotes; ROS-associated activity against intracellular amastigotes	Mechanistic *in vitro* evidence in promastigotes and intracellular amastigotes	The proposed mechanism was investigated in both parasite stages, but direct molecular-target validation and *in vivo* pharmacological characterization remain limited	Fonseca-Silva et al., 2011
Holanamine	Steroidal alkaloid from *Holarrhena pubescens*	*L. donovani*	Enzyme assays, promastigotes, intracellular amastigotes, and host-cell cytotoxicity assays	Inhibition of DNA topoisomerase IB, oxidative and mitochondrial damage in promastigotes, and clearance of intracellular amastigotes	Mechanistic cellular evidence	Intracellular activity and host-cell selectivity were demonstrated, but *in vivo* efficacy, pharmacokinetics, and long-term safety remain untested	Goel et al., 2023
Ursolic acid	Pentacyclic triterpenoid	*L. amazonensis*	Promastigotes, intracellular amastigotes and infected BALB/c mice	Mitochondria-associated programmed-cell-death-like changes, NO production and reduced parasite burden	Preclinical *in vivo*	Pharmacokinetics, formulation and clinical safety remain undefined	Yamamoto et al., 2015
β-Sitosterol	Plant-derived phytosterol from *Corchorus capsularis*	*L. donovani*	Biochemical and cellular assays	Inhibition of trypanothione reductase and disruption of parasite redox homeostasis	Mechanistic preclinical	Target engagement and therapeutic exposure require further validation	Pramanik et al., 2020
Berberine-loaded liposomes	Isoquinoline alkaloid in a liposomal formulation	*L. infantum*	*In-vitro* assays and infected BALB/c mice	Increased selectivity and tissue accumulation and greater reduction of parasite burden relative to free berberine	Formulation-based preclinical	The comparative advantage was formulation-specific; complete parasite elimination, sustained post-treatment efficacy, long-term safety, scalability, and clinical pharmacokinetics remain unresolved	Calvo et al., 2020
Curcumin	Polyphenol from *Curcuma longa*	*L. major*	Infected human PBMCs	Increased IFN-γ, TNF-α and iNOS expression	Host-response evidence	Cytokine changes alone do not establish therapeutic parasite clearance	Alinejad et al., 2022
Fisetin and luteolin	Flavonoids	*L. amazonensis*	Recombinant-arginase inhibition and molecular-modeling analyses	Inhibition of parasite arginase	Preliminary biochemical and computational evidence	Enzyme inhibition and molecular modeling do not establish activity against intracellular amastigotes or intracellular target engagement	Manjolin et al., 2013
Phenolic arginase inhibitors	Naturally occurring phenolic compounds	*L. infantum*	Recombinant-arginase, promastigote, intracellular-amastigote, and macrophage-cytotoxicity assays	Inhibition of recombinant parasite arginase and activity against promastigotes and intracellular amastigotes	Biochemical and intracellular cellular evidence	Intracellular activity and selectivity were demonstrated, but direct evidence that arginase inhibition mediates parasite killing and *in vivo* efficacy remain limited	Garcia et al., 2019
Flavonoids from *Kalanchoe pinnata*	Plant-derived flavonoids	*Leishmania spp*.	Intracellular *L. amazonensis* amastigote assay	Direct antiparasitic activity; mechanism incompletely established	Intracellular *in vitro* evidence	The molecular mechanism, direct target, pharmacokinetics, and efficacy *in vivo* were not established in the cited study.	Muzitano et al., 2006

### 4.3 Mechanisms of action of natural products

Natural products exert antileishmanial activity through diverse and, in some cases, complementary mechanisms directed against the parasite and the infected host cell. Experimentally supported parasite-directed mechanisms include oxidative stress, mitochondrial dysfunction, interference with redox homeostasis, inhibition of parasite-specific enzymes and disruption of DNA replication. However, the strength of this evidence varies considerably among compounds. Mechanistic conclusions based exclusively on promastigote assays should be interpreted cautiously because promastigotes differ metabolically and biologically from the intracellular amastigote stage responsible for mammalian infection.

Mitochondrial dysfunction and oxidative stress are among the most frequently reported mechanisms. Quercetin increased reactive oxygen species (ROS), reduced mitochondrial membrane potential and induced death in *L. amazonensis* promastigotes (Fonseca-Silva et al., 2011). A subsequent study demonstrated that quercetin also induced ROS-dependent death of intracellular amastigotes, providing evidence in a more clinically relevant parasite stage (Fonseca-Silva et al., 2013). Apigenin similarly induced ROS production and (Fonseca-Silva et al., 2015). Importantly, orally administered apigenin reduced lesion development and parasite burden in experimentally infected mice; its activity against intracellular amastigotes was associated with ROS production and autophagic processes (Fonseca-Silva et al., 2016). An orally active clerodane diterpene, K-09, also caused mitochondrial depolarization, ATP depletion, oxidative stress, DNA fragmentation and apoptosis-like death in *L. donovani* promastigotes (Kathuria et al., 2014). These findings support mitochondrial and redox disruption as recurrent mechanisms, although the predominance of promastigote-based experiments remains an important limitation.

Some natural compounds act against defined parasite enzymes. Holanamine, a steroidal alkaloid isolated from *Holarrhena pubescens*, inhibited the heterodimeric DNA topoisomerase IB of *L. donovani*, resulting in impaired parasite growth (Goel et al., 2023). β-Sitosterol derived from *Corchorus capsularis* inhibited trypanothione reductase, disrupted thiol-dependent redox homeostasis and induced oxidative stress in *L. donovani* (Pramanik et al., 2020). Niranthin, a lignan isolated from *Phyllanthus amarus*, stabilized DNA-topoisomerase IB cleavage complexes and inhibited the relegation of cleaved DNA strands (Chowdhury et al., 2012). Unlike many preliminary mechanistic studies, niranthin was active against intracellular amastigotes and antimony-resistant parasites. Treatment of infected BALB/c mice substantially reduced hepatic and splenic parasite burdens and was associated with increased nitric oxide and Th1 cytokine production. This study therefore provides relatively strong evidence for simultaneous parasite-directed and host-mediated activity.

Host immune modulation represents another relevant mechanism. Curcumin treatment of *L. major*-infected peripheral blood mononuclear cells increased ROS generation and expression of IFN-γ, TNF-α and iNOS (Alinejad et al., 2022). Nevertheless, these *ex-vivo* changes do not by themselves demonstrate parasite clearance or clinical efficacy. Ursolic acid provides stronger preclinical evidence: it reduced intracellular *L. amazonensis* amastigotes and parasite burden in infected BALB/c mice, with activity associated with nitric oxide production and programmed-cell-death-like alterations in the parasite (Yamamoto et al., 2015). Glycyrrhizic acid has also been shown to suppress parasite-induced cyclooxygenase-2-mediated anti-inflammatory responses during experimental *L. donovani* infection, thereby promoting a more protective immune environment (Bhattacharjee et al., 2012). Collectively, these findings indicate that natural compounds may influence macrophage activation, nitric oxide production and Th1/Th2 balance, but such effects are compound- and model-dependent and should not be generalized to all phytochemicals.

Formulation and combination strategies can further influence biological activity. Compared with free berberine, berberine-loaded liposomes increased drug accumulation in the liver and spleen and produced greater reductions in tissue parasite burden in *L. infantum*-infected BALB/c mice (Calvo et al., 2020). However, this advantage was demonstrated only for the specific liposomal formulation and experimental conditions tested, and the study did not establish complete parasite elimination, sustained post-treatment activity, or long-term formulation safety. In experimental cutaneous leishmaniasis, apigenin and miltefosine demonstrated an additive interaction *in vitro* (ΣFIC = 1.61), while a reduced-dose combination decreased lesion size and parasite burden in infected mice (Emiliano and Almeida-Amaral, 2018). These findings support a potentially useful combined effect but do not demonstrate synergism. Although this provides evidence that selected natural compounds may function as adjuncts to conventional therapy, synergism, sustained post-treatment efficacy, and protection against relapse must be established experimentally for each combination.

Overall, available evidence supports several mechanistic classes, including mitochondrial and redox disruption, inhibition of parasite-specific enzymes, modulation of macrophage antimicrobial activity and improved intracellular drug delivery. Nevertheless, only a limited number of studies integrate molecular-target validation with intracellular amastigote assays and efficacy *in vivo*. Consequently, claims that natural products are intrinsically multitargeted, less susceptible to resistance or universally synergistic with conventional drugs remain premature.

## 5 Natural products targeting parasite-specific pathways

Natural products can interfere with biochemical processes required for *Leishmania* survival, including thiol-dependent redox defense, polyamine metabolism, mitochondrial function and DNA replication. Some of these pathways contain parasite enzymes that differ structurally or functionally from their mammalian counterparts and may therefore offer opportunities for selective inhibition. However, evidence for reduced resistance or broad multitarget activity remains limited and should be established separately for each compound.

### 5.1 Targeting redox balance and antioxidant defenses

Activated macrophages restrict intracellular *Leishmania* through reactive oxygen species (ROS) and reactive nitrogen species, particularly nitric oxide. To survive this oxidative environment, the parasite relies on a trypanothione-centered antioxidant system rather than the glutathione reductase system used by mammalian cells. Reduced trypanothione supplies reducing equivalents to several antioxidant enzymes and is regenerated from trypanothione disulfide by trypanothione reductase. Disruption of this pathway increases parasite susceptibility to oxidative damage and makes trypanothione reductase an attractive parasite-selective target (Khan, 2007).

Direct experimental support is provided by β-sitosterol isolated from *Corchorus capsularis*. β-Sitosterol inhibited *L. donovani* trypanothione reductase and altered parasite redox homeostasis, leading to ROS accumulation, mitochondrial depolarization and apoptosis-like cellular changes (Pramanik et al., 2020). Nevertheless, this evidence remains preclinical, and selective target engagement in infected animals has not yet been established. Flavonoids can also disturb parasite redox balance through mechanisms that may extend beyond direct trypanothione reductase inhibition. Quercetin increased ROS production and caused mitochondrial dysfunction in *L. amazonensis* promastigotes. Importantly, a subsequent study demonstrated ROS-dependent killing of intracellular amastigotes, strengthening the biological relevance of this mechanism (Fonseca-Silva et al., 2013).

Other natural compounds similarly generate oxidative stress through mitochondrial or redox disruption. Apigenin induced ROS accumulation and loss of mitochondrial membrane potential in *L. amazonensis* (Fonseca-Silva et al., 2015). Oral apigenin also reduced lesion size and parasite burden in infected mice, with its activity associated with ROS production and autophagic processes in intracellular amastigotes (Fonseca-Silva et al., 2016). In *L. donovani*, the clerodane diterpene K-09 produced mitochondrial depolarization, decreased ATP levels, increased ROS, and induced DNA fragmentation and apoptosis-like death (Kathuria et al., 2014). Curcumin has likewise been reported to induce ROS accumulation, calcium imbalance, mitochondrial dysfunction and programmed-cell-death-like changes in *L. donovani* (Das et al., 2008). Quinones such as lapachol and plumbagin may undergo redox cycling and promote oxidative stress in *Leishmania* (Ali et al., 2010).

Collectively, these findings support redox disruption as a recurring antileishmanial mechanism. Nevertheless, increased ROS and mitochondrial depolarization are frequently downstream indicators of parasite injury rather than evidence of a defined molecular target. Future studies should distinguish direct inhibition of redox enzymes from nonspecific oxidative damage and confirm activity against intracellular amastigotes, resistant clinical isolates and mammalian infection models.

### 5.2 Inhibition of arginase and polyamine metabolism

The arginase–polyamine pathway contributes to *Leishmania* growth, differentiation and resistance to oxidative stress. Parasite arginase hydrolyses L-arginine to ornithine, which is subsequently used to synthesize putrescine and spermidine. Spermidine is also required for trypanothione synthesis, linking polyamine metabolism to the parasite antioxidant defense system (Pessenda and Da Silva, 2020). Altered expression of polyamine-pathway components has been detected in lesions and plasma from patients with diffuse cutaneous leishmaniasis, indicating that this metabolic axis is perturbed during human disease (Malta-Santos et al., 2020). However, these clinical associations do not establish that arginase activity is directly responsible for disease progression.

Although genetic and biochemical findings support parasite arginase as a potential therapeutic target, its essentiality is context-dependent. Arginase-deficient parasites display impaired polyamine biosynthesis but can retain some infectivity by salvaging ornithine or polyamines from the host environment (Pessenda and Da Silva, 2020). Therefore, inhibition of parasite arginase may suppress parasite growth without necessarily producing complete clearance. This metabolic plasticity represents an important limitation and suggests that arginase inhibition may be more effective when combined with interventions targeting polyamine transport, downstream biosynthetic enzymes or trypanothione metabolism.

Several natural compounds inhibit recombinant *Leishmania* arginase. Fisetin, luteolin and related flavonoids inhibited *L. amazonensis* arginase and reduced parasite growth in culture. Enzyme-inhibition and molecular-modeling analyses suggested interactions with residues located within or near the enzyme's catalytic region (Manjolin et al., 2013). However, the study did not demonstrate that arginase inhibition was responsible for parasite death in intracellular amastigotes or infected animals. Thus, the findings establish biochemical activity but provide limited evidence of intracellular target engagement.

Naturally occurring phenolic compounds, including caffeic acid and rosmarinic acid, also inhibited recombinant *L. infantum* arginase (Garcia et al., 2019). Although these compounds showed measurable enzyme-inhibitory activity, their cellular uptake, metabolic stability and selectivity for parasite over mammalian arginases remain incompletely defined. Enzyme inhibition alone is insufficient to establish therapeutic potential because compounds may fail to reach the parasite enzyme within the parasitophorous vacuole or may affect host arginine metabolism at pharmacologically relevant concentrations.

Green-tea flavanols provide additional enzyme-level evidence. EGCG, (+)-catechin and (–)-epicatechin inhibited recombinant *L. amazonensis* arginase, with (+)-catechin demonstrating the greatest potency. Kinetic analyses identified EGCG as a mixed inhibitor, whereas catechin and epicatechin acted predominantly as competitive inhibitors (Dos Reis et al., 2013). Although these flavanols were more active against parasite arginase than rat liver arginase, they also inhibited the mammalian enzyme. Therefore, relative biochemical potency should not be interpreted as complete parasite selectivity, and potential effects on host arginine metabolism require further investigation.

Verbascoside provides stronger evidence connecting arginase inhibition with antileishmanial activity. It inhibited *L. amazonensis* arginase and suppressed promastigote growth (Maquiaveli et al., 2016). A subsequent study demonstrated activity against intracellular amastigotes and used L-arginine supplementation and macrophage-activation conditions to support parasite arginase as a functionally relevant target (Maquiaveli et al., 2017). This represents more biologically meaningful evidence than recombinant-enzyme inhibition alone. Nevertheless, the findings remain limited to cellular models, and direct target engagement, pharmacokinetics, toxicity and therapeutic efficacy in infected animals have not been established.

Computational predictions should be clearly distinguished from experimentally validated inhibition. Δ9-Tetrahydrocannabinol, cannabidiol and caryophyllene oxide from *Cannabis sativa* were predicted to interact with both parasite and human arginases (Assouab et al., 2022). Because this study relied on molecular docking without enzyme-inhibition, intracellular-amastigote or animal experiments, these compounds should be considered candidates for biochemical validation rather than established arginase-targeting antileishmanial agents.

Arginine metabolism also influences host antimicrobial immunity. Within macrophages, host arginase and inducible nitric oxide synthase compete for L-arginine. Increased host arginase activity can favor ornithine and polyamine production, whereas iNOS-dependent metabolism generates nitric oxide required for intracellular parasite killing (Pessenda and Da Silva, 2020). However, inhibition of parasite arginase should not automatically be assumed to increase L-arginine availability for host iNOS because the parasite and host enzymes are located in distinct cellular compartments. Natural compounds proposed to exert both metabolic and immunological effects must therefore be evaluated independently for parasite-arginase inhibition, host-arginase modulation, nitric oxide production and intracellular parasite clearance.

Overall, the arginase–polyamine pathway is supported by biochemical and cellular evidence as a promising but metabolically bypassable antileishmanial target. The strongest natural-product evidence currently concerns fisetin, luteolin, green-tea flavanols, naturally occurring phenolic acids and verbascoside. However, most studies remain at the enzyme-screening or *in vitro* stage. Clinical translation will require demonstration of selectivity over mammalian arginases, intracellular target engagement, activity against clinical amastigote isolates, evaluation of polyamine-salvage mechanisms and therapeutic efficacy in suitable animal models.

### 5.3 Interference with parasite signaling and replication

Natural products can interfere with parasite replication by disrupting DNA synthesis, DNA topology, cell-cycle progression and mitochondrial homeostasis. However, these mechanisms are not supported equally. Direct inhibition of a purified parasite enzyme constitutes stronger target-level evidence than molecular docking, changes in gene expression or downstream markers such as ROS accumulation, mitochondrial depolarization and DNA fragmentation.

DNA topoisomerase IB is a particularly attractive target because *Leishmania* possesses a heterodimeric enzyme that differs structurally from the monomeric topoisomerase I found in mammalian cells. Holanamine, a steroidal alkaloid isolated from *Holarrhena pubescens*, inhibited *L. donovani* DNA topoisomerase IB and stabilized enzyme–DNA cleavage complexes, thereby interfering with DNA-relaxation activity. Holanamine also inhibited intracellular amastigotes, induced ROS production and apoptosis-like changes, and reduced parasite burden in infected animals (Goel et al., 2023). These findings support topoisomerase IB as a functionally relevant target, although pharmacokinetic behavior, selectivity over human topoisomerases and long-term toxicity remain to be established.

Niranthin provides further evidence linking topoisomerase inhibition with antileishmanial efficacy. This lignan inhibited the relaxation activity of heterodimeric *L. donovani* topoisomerase IB, promoted topoisomerase-mediated DNA–protein adduct formation and induced DNA fragmentation. It was active against intracellular amastigotes and antimony-resistant parasites and reduced hepatic and splenic parasite burdens in infected BALB/c mice (Chowdhury et al., 2012). Niranthin treatment was also associated with increased nitric oxide and Th1 cytokine production, suggesting combined parasite-directed and host-mediated activity. Nevertheless, the relative contribution of topoisomerase inhibition and immune modulation to the *in vivo* response remains unresolved.

Natural compounds may also impair DNA synthesis through effects on nucleotide metabolism. Quercetin interfered with iron metabolism in *L. donovani* and reduced the activity of the iron-dependent enzyme ribonucleotide reductase, which catalyzes a rate-limiting step in deoxyribonucleotide synthesis. Quercetin combined with serum albumin reduced splenic parasite burden more effectively than unformulated quercetin in an experimental infection model (Sen et al., 2008). This study provides *in vivo* evidences connecting altered iron availability with impaired DNA synthesis. However, quercetin is pharmacologically pleiotropic, and ribonucleotide-reductase inhibition may occur alongside oxidative and mitochondrial effects rather than representing its only mechanism.

Mitochondrial dysfunction is another frequently reported consequence of natural-product treatment. Quercetin increased ROS production and caused loss of mitochondrial membrane potential in *L. amazonensis* promastigotes (Fonseca-Silva et al., 2011). Curcumin similarly induced ROS accumulation, disturbed calcium homeostasis and produced mitochondrial and programmed-cell-death-like alterations in *L. donovani* (Das et al., 2008). Vanillin-derived compounds have also been reported to induce oxidative stress, mitochondrial depolarization and morphological changes in cutaneous and visceral *Leishmania* species (Freitas et al., 2023). These observations demonstrate parasite injury but do not necessarily identify the mitochondrion as the compound's primary molecular target. ROS accumulation and mitochondrial depolarization can arise downstream of damage to DNA, redox enzymes or other cellular components.

The term “apoptosis-like death” should also be used cautiously. *Leishmania* can exhibit phosphatidylserine exposure, DNA fragmentation, cell shrinkage, ROS accumulation and mitochondrial depolarization after compound treatment. However, the parasite lacks the canonical mammalian caspase-dependent apoptotic machinery. These phenotypes therefore describe a programmed-cell-death-like process and should not be interpreted as evidence that mammalian apoptosis pathways operate unchanged in the parasite.

Evidence for direct regulation of parasite kinase signaling by natural products remains comparatively weak. Changes in phosphorylation, calcium balance or stress-responsive gene expression may indicate disrupted signaling, but they do not establish direct binding to a specific kinase or signaling protein. Molecular docking and expression profiling should therefore be described as hypothesis-generating unless supported by biochemical inhibition, genetic validation or target-rescue experiments.

**Figure 2:**
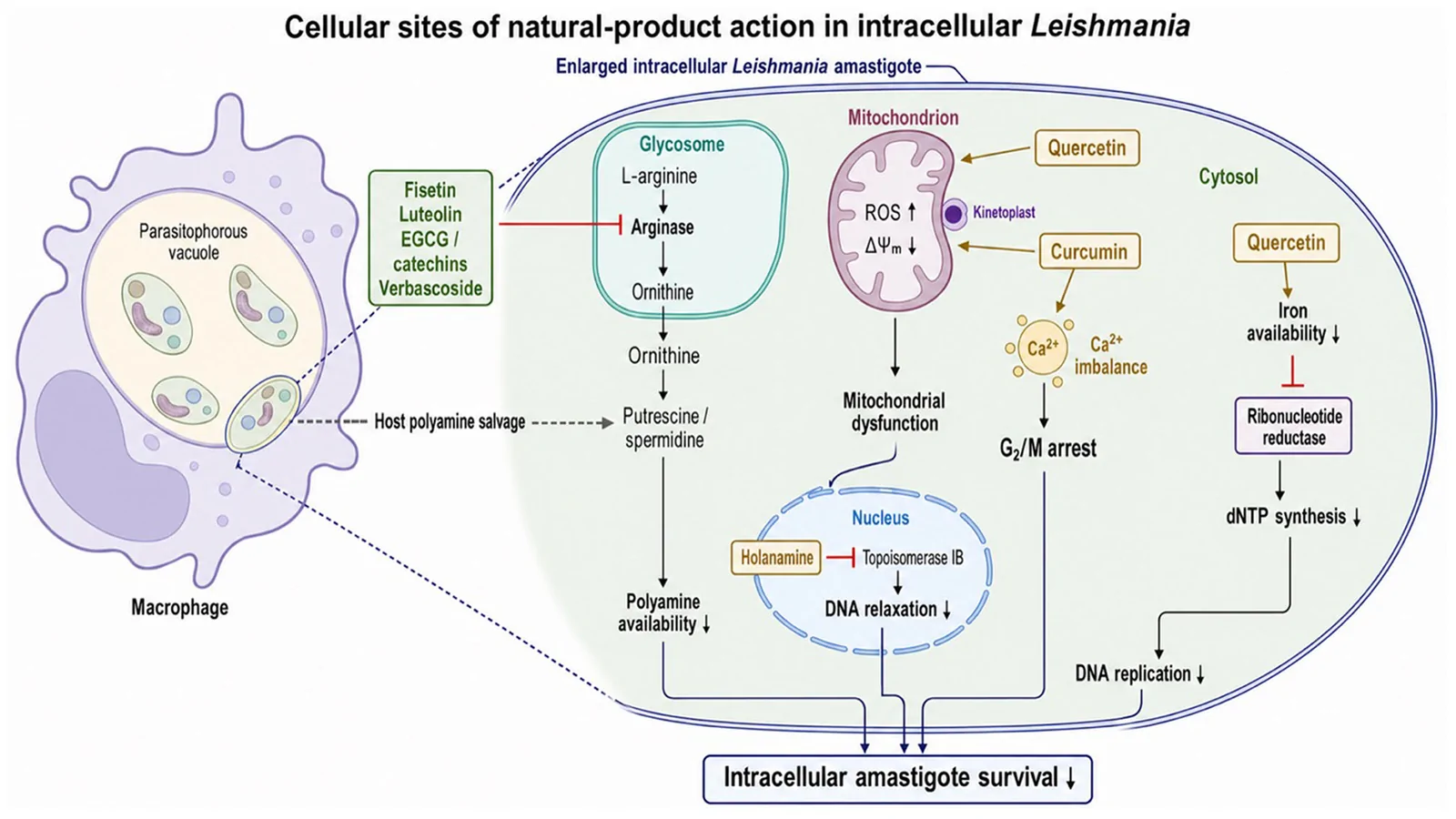
Cellular sites of natural-product action in intracellular *Leishmania*. Within macrophage parasitophorous vacuoles, natural compounds affect distinct metabolic and cellular processes in intracellular amastigotes. Fisetin and luteolin inhibit parasite arginase in biochemical and parasite-culture models (Manjolin et al., 2013), whereas EGCG and related catechins inhibit recombinant *L. amazonensis* arginase but also show activity against mammalian arginase (Dos Reis et al., 2013). Verbascoside provides additional evidence linking arginase inhibition with activity against promastigotes and intracellular amastigotes (Maquiaveli et al., 2016, 2017). The dashed polyamine-salvage route represents potential metabolic compensation rather than a demonstrated consequence of compound treatment (Roberts et al., 2004). Quercetin induces ROS accumulation and mitochondrial dysfunction in *L. amazonensis* (Fonseca-Silva et al., 2011), while curcumin disrupts ROS and Ca^2^^+^ homeostasis and induces G_2_/M arrest in *L. donovani* promastigotes (Das et al., 2008). Holanamine inhibits *L. donovani* topoisomerase IB and reduces promastigote and intracellular-amastigote survival (Goel et al., 2023). Quercetin also interferes with iron-dependent ribonucleotide reductase and DNA synthesis, with reduced splenic parasite burden reported using a quercetin–serum albumin formulation (Sen et al., 2008). Arrows indicate experimentally observed or biologically supported consequences; the dashed arrow indicates a compensatory route.

Overall, topoisomerase IB inhibition by holanamine and niranthin currently provides the strongest target-specific evidence in this section. Quercetin-mediated disruption of iron-dependent ribonucleotide reductase provides an additional replication-related mechanism supported by animal data. By contrast, mitochondrial depolarization, ROS accumulation and apoptosis-like phenotypes are common downstream responses whose initiating targets frequently remain uncertain. Future studies should integrate biochemical target validation, resistant-mutant or genetic-rescue approaches, intracellular-amastigote assays and pharmacologically relevant animal models to distinguish primary mechanisms from secondary cellular injury. The cellular localization of these experimentally supported natural-product targets—including parasite arginase, mitochondrial and calcium homeostasis, topoisomerase IB and ribonucleotide reductase—is summarized in Figure 2.

## 6 Immunomodulatory effects of natural products in leishmaniasis

Leishmaniasis remains a substantial global health burden, particularly in resource-limited regions. The World Health Organization estimates that approximately 600,000–1 million new cases of cutaneous leishmaniasis and 50,000–90,000 cases of visceral leishmaniasis occur annually, although incomplete surveillance means that the true burden is probably underestimated (Alvar et al., 2012). Disease outcome is determined not only by parasite replication but also by the quality and regulation of the host immune response. IFN-γ-dependent macrophage activation generally promotes parasite control, whereas IL-10, TGF-β and alternatively activated macrophage programmes can facilitate intracellular persistence. However, the conventional Th1-protective/Th2-susceptible framework is an oversimplification because excessive inflammatory responses may also contribute to tissue damage, particularly in cutaneous and mucosal disease (Scott and Novais, 2016).

Current chemotherapy relies primarily on pentavalent antimonials, amphotericin B, miltefosine, paromomycin and pentamidine. Their utility is limited by toxicity, route and duration of administration, variable species- and region-dependent efficacy, treatment failure and emerging resistance (Croft et al., 2006; Croft and Olliaro, 2011; Sundar and Chakravarty, 2015). These limitations have encouraged investigation of natural products that may combine direct antiparasitic activity with modulation of host immunity (Chouhan et al., 2014; Seth and Kar, 2023).

Experimental studies suggest that flavonoids, alkaloids, terpenoids, polyphenols and plant-derived fractions can modify macrophage activation, cytokine production and nitric-oxide-dependent parasite killing (Chouhan et al., 2014). Nevertheless, immunomodulatory activity should not be inferred solely from a reduction in parasite burden. A compound can reduce infection through direct parasite toxicity, altered host-cell viability or genuine immune activation, and these mechanisms require separate experimental assessment. Strong evidence for host-directed activity should therefore include infected-cell or animal studies demonstrating immune changes alongside parasite reduction, preferably supported by pathway inhibition, cytokine neutralization or genetic validation.

The following subsections critically examine evidence for natural products that influence macrophage activation, Th1-associated responses, nitric oxide production and parasite-permissive immune programmes. Particular attention is given to whether the reported immune changes are mechanistically linked to parasite clearance and whether the findings have been confirmed beyond promastigote or isolated-cell models.

### 6.1 Natural products shaping host immune responses

The outcome of *Leishmania* infection is influenced by coordinated interactions among macrophages, dendritic cells and different T-cell populations. IL-12-dependent Th1 responses promote IFN-γ production and classical macrophage activation, resulting in inducible nitric oxide synthase (iNOS) expression and the production of nitric oxide and reactive oxygen species that contribute to intracellular parasite killing (Nathan and Shiloh, 2000; Olivier et al., 2005). Conversely, IL-10, TGF-β and alternatively activated macrophage programmes can suppress microbicidal activity and support parasite persistence (Nylén and Sacks, 2007; Scott and Novais, 2016).

Several natural products have been reported to increase Th1-associated cytokines, stimulate nitric oxide production or reduce parasite-permissive cytokines, suggesting potential host-directed antileishmanial activity (Chouhan et al., 2014; Seth and Kar, 2023). However, changes in cytokine concentrations alone do not establish that immune modulation is responsible for parasite clearance. Reduced parasite numbers may decrease immunosuppressive signaling secondarily, while direct compound toxicity may occur independently of macrophage activation. Mechanistic claims therefore require experiments showing that inhibition or neutralization of the proposed immune pathway reverses the antileishmanial effect.

The commonly used Th1/Th2 framework also requires careful interpretation. Although Th1-associated responses are generally protective, excessive IFN-γ, TNF-α and cytotoxic-cell activity can contribute to inflammatory tissue injury in some forms of cutaneous and mucosal leishmaniasis. In addition, IL-10 may be produced by regulatory T cells, macrophages and Th1 cells rather than exclusively reflecting a Th2 response (Nylén and Sacks, 2007; Scott and Novais, 2016). Th17 responses are similarly context-dependent and may support parasite control or exacerbate inflammation according to the infecting species, tissue and disease stage.

Natural compounds should therefore not be evaluated solely by their capacity to increase the Th1/Th2 ratio. More informative endpoints include intracellular amastigote burden, macrophage iNOS and nitric oxide production, cytokine source, immune-cell phenotype and evidence that blocking the affected pathway alters compound efficacy. These criteria are used in the following subsections to distinguish experimentally supported immunomodulation from descriptive cytokine associations.

#### 6.1.1 Enhancement of Th1 responses

IL-12 produced mainly by dendritic cells and macrophages promotes Th1 differentiation and stimulates natural killer and T cells to secrete IFN-γ. IFN-γ subsequently activates infected macrophages and enhances iNOS- and NADPH-oxidase-dependent antimicrobial responses (Nathan and Shiloh, 2000; Seth and Kar, 2023). Natural products that increase IL-12 or IFN-γ may therefore support parasite control. However, cytokine induction should be considered mechanistically relevant only when it is accompanied by reduced intracellular amastigote burden and when disruption of the proposed immune pathway diminishes the antileishmanial effect.

Berberine has been widely reported to regulate NF-κB, JAK/STAT and MAPK signaling in different inflammatory models (Haftcheshmeh et al., 2022). Liposome-encapsulated berberine also reduced hepatic and splenic parasite burdens in *L. infantum*-infected BALB/c mice (Calvo et al., 2020). Nevertheless, that efficacy study did not establish that Th1 activation was responsible for parasite clearance. The formulation could have improved drug delivery to infected macrophages and increased direct parasite exposure. Therefore, berberine should be described as an *in vivo* active antileishmanial compound with potential immunomodulatory properties, rather than as a confirmed Th1-enhancing therapy.

Curcumin provides infected-cell evidence of immune-associated activity. In *L. major*-infected human peripheral blood mononuclear cells, curcumin increased IFN-γ, TNF-α and iNOS gene expression in a concentration- and time-dependent manner (Alinejad et al., 2022). This finding is consistent with activation of a macrophage-supportive inflammatory response but remains based primarily on transcriptional measurements. Cytokine protein secretion, nitric oxide production, intracellular amastigote killing and pathway-blocking experiments were not comprehensively demonstrated. Moreover, curcumin can exert anti-inflammatory effects in other experimental settings, indicating that its immunological action depends on concentration, formulation, cell type and inflammatory context (Alinejad et al., 2022; Dourado et al., 2024).

Mahanine provides stronger evidence because its activity has been investigated in both infected cells and animals. Mahanine suppressed parasite-associated uncoupling protein 2, restored oxidative responses and increased NO, ROS, iNOS and IL-12-associated activity. Oral treatment also reduced hepatic and splenic parasite burdens in *L. donovani*-infected mice (Roy et al., 2017). These findings support combined parasite-directed and host-mediated activity. Nevertheless, the relative contributions of direct parasite toxicity, restoration of macrophage oxidative function and adaptive Th1 activation were not completely separated.

Niranthin similarly combines a defined parasite target with host immune effects. It inhibited *L. donovani* topoisomerase IB and reduced parasite survival, while treatment of infected mice increased IL-12- and IFN-γ-associated responses and promoted NO-dependent macrophage activation (Roy et al., 2017). Because both direct topoisomerase inhibition and immune changes occurred, the reduction in parasite burden cannot be attributed exclusively to Th1 polarization. Niranthin is therefore better classified as a dual-action compound than as a purely host-directed immunomodulator.

Evidence for polysaccharide- and tannin-enriched plant fractions is less conclusive. Although some preparations reportedly increase IL-12, IFN-γ or lymphocyte proliferation, heterogeneous chemical composition and limited receptor-validation experiments make it difficult to assign these effects to a specific constituent or signaling pathway (Chouhan et al., 2014). Claims involving TLR4, Dectin-1 or other pattern-recognition receptors require receptor-blocking or genetic studies in infected cells. Overall, mahanine and niranthin currently provide stronger integrated cellular and animal evidence, whereas the Th1-mediated contributions of berberine, curcumin and complex plant fractions require further mechanistic validation.

#### 6.1.2 Augmentation of nitric oxide production

Inducible nitric oxide synthase (iNOS/NOS2) generates nitric oxide in activated macrophages and contributes to intracellular *Leishmania* killing through modification of parasite proteins, disruption of mitochondrial metabolism and interaction with other reactive intermediates (Olivier et al., 2005; Bogdan, 2020). The importance of this mechanism is particularly well established in experimental murine leishmaniasis. In human infection, however, the magnitude and contribution of macrophage-derived nitric oxide are more variable and may depend on the *Leishmania* species, tissue environment and activation state of the responding monocytes or macrophages (Carneiro et al., 2016; Bogdan, 2020).

Natural-product studies commonly estimate nitric oxide production using nitrite accumulation in culture supernatants, usually measured by the Griess reaction. Others infer activation of the pathway from increased iNOS mRNA or protein. These measurements are useful but are not interchangeable with demonstration of NO-dependent parasite killing. Bulk nitrite concentrations do not establish the amount of bioactive nitric oxide reaching amastigotes inside parasitophorous vacuoles, while increased iNOS transcription does not necessarily indicate enzymatic activity. Strong mechanistic evidence therefore requires parallel measurement of intracellular amastigote burden and reversal of the compound's activity using an iNOS inhibitor, nitric oxide scavenger or NOS2-deficient model.

A methanolic extract of *Calotropis procera* increased ROS generation in *L. major* promastigotes and upregulated TNF-α, IFN-γ and iNOS transcripts in infected mononuclear cells (Amani et al., 2024). These findings suggest combined parasite-directed and host-cell effects. Nevertheless, ROS generation was demonstrated in extracellular promastigotes, whereas immune-gene expression was measured in infected host cells. The study did not establish that increased iNOS expression produced sufficient nitric oxide to mediate intracellular amastigote killing. Furthermore, the use of a chemically complex plant extract prevents assignment of the activity to a defined constituent.

Diallyl sulfide, an organosulfur constituent of garlic, has also been evaluated in combination with meglumine antimoniate. In *L. major*-infected BALB/c mice, combination treatment increased IFN-γ, TNF-α and nitric-oxide-associated responses and was accompanied by reduced lesion size and parasite burden (Zarrinkar et al., 2024). This provides useful animal-level evidence linking immune activation with therapeutic improvement. However, the study did not use iNOS inhibition or nitric oxide neutralization to demonstrate that NO was necessary for efficacy. Moreover, because diallyl sulfide was administered with meglumine antimoniate, the individual contribution of each agent and whether their interaction was additive or synergistic remain uncertain.

Overall, the available studies show that natural products can increase iNOS expression or nitric-oxide-associated readouts during *Leishmania* infection, but direct evidence that NO mediates their antileishmanial activity remains limited. Future studies should combine intracellular amastigote quantification with iNOS protein and activity measurements, appropriate nitric oxide controls and pathway-blocking experiments. Species- and tissue-specific validation is also necessary before NO induction can be treated as a generally reliable predictor of therapeutic efficacy.

#### 6.1.3 Suppression of parasite-favorable immune responses

*Leishmania* persistence is supported by regulatory cytokines and alternatively activated macrophage programmes that suppress intracellular killing. IL-10 and TGF-β inhibit macrophage activation and can increase host arginase-1 expression, diverting L-arginine metabolism away from iNOS-dependent nitric oxide production and toward ornithine and polyamine synthesis (Iniesta et al., 2005; Nylén and Sacks, 2007; Kupani et al., 2021). IL-4 and IL-13 can additionally promote STAT6-dependent alternative macrophage activation. However, these responses should not collectively be described as “Th2 immunity,” because IL-10 and TGF-β may be produced by regulatory T cells, macrophages and even chronically activated Th1 cells.

Prostaglandin E_2_ represents another parasite-permissive host pathway. In *L. donovani*-infected macrophages, PGE_2_ signaling through the EP2 receptor increased cAMP-dependent signaling and suppressed inflammatory mediators, whereas EP2 antagonism reduced hepatic and splenic parasite burdens in infected animals (Saha et al., 2014). This study validates EP2-associated signaling as a host-directed target but does not demonstrate that natural products act through this pathway. Although several polyphenols can inhibit cyclooxygenase activity in other inflammatory models, their ability to suppress infection-associated PGE_2_-EP2 signaling must be tested directly before this mechanism can be assigned to antileishmanial natural products.

Berberine has been reported to regulate NF-κB, JAK/STAT and MAPK pathways and to suppress IL-10-associated inflammatory programmes in non-leishmanial systems (Haftcheshmeh et al., 2022). Nevertheless, convincing compound-specific evidence that berberine reverses IL-10- or STAT6-dependent macrophage polarization during active *Leishmania* infection remains limited. Reduced IL-10 after treatment may also result secondarily from decreased parasite burden rather than represent the initiating mechanism of parasite clearance. Experiments using IL-10 receptor blockade, STAT6 inhibition or macrophage-polarization markers would be needed to resolve this direction of causality.

Host arginase-1 is a potentially important metabolic target because its induction is associated with impaired nitric oxide production in human visceral leishmaniasis (Kupani et al., 2021). This host enzyme must be distinguished from parasite arginase, which is localized within the parasite glycosome and supports parasite polyamine biosynthesis. Natural compounds that inhibit recombinant parasite arginase cannot therefore be assumed to inhibit macrophage arginase-1 or restore host iNOS activity. The two effects require independent enzyme-selectivity and infected-macrophage experiments.

Overall, evidence that natural products directly reverse parasite-permissive immune programmes is less developed than evidence for their effects on parasite growth or general cytokine profiles. Strong validation would require simultaneous measurement of intracellular amastigote burden, host arginase-1 activity, iNOS/NO production and the cellular sources of IL-10 and TGF-β. Pathway-blocking experiments are also necessary to demonstrate that suppression of these regulatory mechanisms is required for treatment efficacy. This distinction is particularly important in visceral leishmaniasis, where IL-10-mediated immune suppression is well documented but differs substantially among circulating monocytes, hepatic macrophages and splenic macrophage populations (Nylén and Sacks, 2007; Kupani et al., 2021; Kaye and Aebischer, 2011).

#### 6.1.4 Natural products and host immunometabolism

Host-cell metabolism is increasingly recognized as an important determinant of *Leishmania* persistence. In *L. infantum*-infected macrophages, an early increase in glycolysis is followed by a shift toward mitochondrial oxidative metabolism. This later metabolic state depends partly on the host SIRT1–LKB1–AMPK axis and supports intracellular parasite survival. Genetic or pharmacological disruption of this pathway reduced parasite burden in infected cells and animals, providing direct evidence that host macrophage metabolism can be therapeutically targeted (Moreira et al., 2015).

A related study showed that *L. donovani* promotes macrophage mitochondrial biogenesis and oxidative phosphorylation through parasite lipophosphoglycan, host Toll-like receptors and type-I-interferon signaling. Pharmacological induction of mitochondrial biogenesis increased macrophage permissiveness to parasite replication, supporting the biological relevance of this metabolic adaptation (Acevedo Ospina et al., 2022). These findings also demonstrate that macrophage activation cannot be represented by a fixed glycolytic-M1 versus oxidative-M2 division: metabolic changes vary with infection stage, parasite species and host-cell context.

Berberine, curcumin and quercetin regulate AMPK, mTOR, HIF-1α, mitochondrial activity or lipid metabolism in cancer, metabolic-disease and non-infectious inflammatory models. However, these observations cannot be directly extrapolated to *Leishmania*-infected macrophages. No convincing study has yet demonstrated that the antileishmanial activity of these natural products depends on correction of parasite-induced host metabolic reprogramming. Their effects on macrophage glycolysis, oxidative phosphorylation, mitochondrial biogenesis and metabolic flux during infection remain largely uncharacterized.

Consequently, host immunometabolism should currently be presented as an evidence-supported therapeutic opportunity but an unvalidated mechanism for most natural products. Future investigations should combine intracellular amastigote quantification with extracellular-flux analysis, isotope-tracing metabolomics and measurements of AMPK, SIRT1, mTOR, HIF-1α and PGC-1α activity. Pathway inhibition or macrophage-specific genetic models will be required to determine whether metabolic changes are necessary for compound efficacy rather than secondary consequences of reduced infection.

### 6.2 Dual action of natural compounds

Natural products may offer therapeutic value by combining direct activity against *Leishmania* with modulation of host-cell responses. Conceptually, such dual activity could reduce dependence on a single parasite target; however, this theoretical advantage should not be interpreted as evidence that a compound prevents or delays resistance. Such claims require direct evaluation using resistant isolates, serial-passage or prolonged drug-pressure experiments, and characterization of the resulting resistance mechanisms (Croft et al., 2006; Croft and Olliaro, 2011; Chouhan et al., 2014). However, a compound should not be classified as dual-acting merely because it reduces parasite burden and changes cytokine concentrations in the same experiment.

Demonstration of direct antiparasitic activity requires evidence from parasite cultures, purified target assays or cell-free biochemical systems. Host-mediated activity requires experiments in infected macrophages or animals showing that modulation of a defined host pathway contributes to parasite reduction. Ideally, blocking the proposed host pathway should diminish efficacy without eliminating the compound's direct parasite activity. Without this separation, immune changes may simply be secondary consequences of reduced parasite burden.

Dual activity may be particularly useful in visceral leishmaniasis, where parasites occupy heterogeneous macrophage populations in the liver, spleen and bone marrow. Nevertheless, the relative contributions of direct and host-mediated mechanisms may vary among tissues because compound exposure, macrophage activation state and parasite susceptibility are not uniform. Evidence obtained from promastigotes or a single macrophage cell line therefore cannot establish dual activity throughout the infected host.

Pharmacokinetics represents another major limitation. Many phytochemicals have low aqueous solubility, rapid metabolism and limited accumulation within macrophage parasitophorous vacuoles. Consequently, the concentration required for direct parasite killing may differ from that required for host immunomodulation, and both concentrations may not be achievable simultaneously *in vivo*. Liposomal and nanoparticle formulations have improved the delivery of berberine and curcumin in experimental systems, but enhanced formulation efficacy does not by itself demonstrate dual action (Calvo et al., 2020; Dourado et al., 2024).

For this review, compounds are considered strongly supported as dual-acting only when parasite-directed activity and host-response modulation have been investigated independently and both are associated with reduced intracellular or tissue parasite burden. Compounds supported only by parallel cytokine and efficacy measurements are classified as putative dual-action candidates. This distinction is applied in the following subsections.

#### 6.2.1 Direct antiparasitic effects

The direct component of dual action refers to compound activity against the parasite that can be demonstrated independently of host immune activation. Evidence may include inhibition of promastigote or axenic-amastigote growth, activity against a purified parasite enzyme, disruption of parasite-specific metabolism or demonstration of parasite damage in the absence of immune effector cells. The principal parasite-directed pathways affected by natural products—including redox homeostasis, arginase–polyamine metabolism and DNA replication—are discussed in detail in Section 5.

Niranthin provides strong evidence of a defined parasite-directed mechanism. It inhibited *L. donovani* topoisomerase IB and interfered with parasite DNA processing, while also showing activity against intracellular parasites and antimony-resistant isolates (Chowdhury et al., 2012). The same study documented Th1-associated immune changes in infected mice, making niranthin a credible dual-action candidate. Nevertheless, direct topoisomerase inhibition and host immune activation were not quantitatively separated *in vivo*, so their respective contributions to tissue parasite clearance remain uncertain.

Mahanine also demonstrates parasite-directed activity associated with disruption of redox homeostasis and mitochondrial function. It increased parasite oxidative stress and produced antileishmanial activity in cellular and murine models (Roy et al., 2017). Because mahanine also affected macrophage oxidative responses and immune-associated mediators, its efficacy may involve both parasite and host pathways. However, the extent to which direct parasite redox disruption accounts for the observed animal efficacy was not determined independently.

Curcumin induces ROS accumulation, calcium imbalance, G_2_/M arrest and apoptosis-like death in *L. donovani* promastigotes, providing host-independent evidence of direct parasite toxicity (Das et al., 2012). Curcumin has also been associated with increased IFN-γ, TNF-α and iNOS expression in infected peripheral blood mononuclear cells (Alinejad et al., 2022). These findings support potential dual action, but the direct and host-directed effects were demonstrated in different experimental systems, using different parasite stages and potentially different concentrations. It has not therefore been established that both mechanisms operate simultaneously at a therapeutically achievable exposure.

Holanamine directly inhibited *L. donovani* topoisomerase IB and reduced promastigote and intracellular-amastigote survival with limited toxicity toward the tested mammalian cells (Goel et al., 2023). This provides strong evidence of parasite-directed activity but not, by itself, of host immunomodulation. Holanamine should therefore be classified as a validated direct-acting compound rather than a dual-action agent unless independent host-response evidence becomes available.

Overall, niranthin and mahanine currently provide the most integrated evidence for combined parasite-directed and host-associated activity. Curcumin remains a plausible dual-action candidate because its two components were demonstrated in separate models, whereas holanamine is better supported as a direct parasite-targeting compound. Future dual-action studies should compare direct parasite potency, intracellular-amastigote activity and host-pathway modulation within the same concentration range and experimental system.

#### 6.2.2 Indirect host-mediated parasite clearance

Indirect host-mediated activity occurs when a compound reduces infection by enhancing macrophage or immune-cell functions rather than solely through direct parasite toxicity. Potential mechanisms include increased IL-12 and IFN-γ signaling, activation of iNOS-dependent nitric oxide production, restoration of macrophage oxidative responses and suppression of IL-10-associated regulatory pathways (Nylén and Sacks, 2007; Chouhan et al., 2014; Scott and Novais, 2016). However, cytokine changes accompanying parasite reduction do not by themselves demonstrate host-mediated killing. The proposed immune pathway must be shown to contribute functionally to efficacy.

Niranthin provides relatively strong evidence of combined host and parasite effects. In addition to inhibiting *L. donovani* topoisomerase IB, treatment of infected mice increased IL-12- and IFN-γ-associated responses and promoted macrophage NO and ROS production (Chowdhury et al., 2012). These immune changes occurred alongside substantial reductions in tissue parasite burden. Nevertheless, because niranthin also directly targets the parasite, pathway-blocking experiments would be required to determine how much of its efficacy *in vivo* depends on immune activation.

Mahanine also produced host-associated changes in infected animals, including increased IL-12, iNOS, NO and ROS responses, while reducing hepatic and splenic parasite burdens (Chowdhury et al., 2012). Suppression of uncoupling protein 2 was proposed to restore macrophage oxidative activity. This provides stronger mechanistic support than cytokine measurement alone, although direct parasite redox disruption also contributes to mahanine activity. It should therefore be considered a dual-action compound rather than a purely host-directed immunomodulator.

Diallyl sulfide combined with meglumine antimoniate increased IFN-γ, TNF-α, nitric-oxide-associated responses and CD4^+^-cell infiltration in *L. major*-infected mice, accompanied by reductions in lesion size and parasite burden (Zarrinkar et al., 2024). Because treatment involved a combination with an established antileishmanial drug, the immune contribution of diallyl sulfide cannot be isolated from the effects of meglumine antimoniate. The absence of formal synergy analysis and immune-pathway blockade further limits assignment of causality.

Synthetic vanillin derivatives showed direct activity against promastigotes and intracellular amastigotes and altered cytokine and nitrite production in infected-cell models (Freitas et al., 2023). These findings identify potentially useful dual-action candidates, but the available study was conducted primarily *in vitro*. Claims that these derivatives generate systemic Th1 responses or reduce parasite burdens in multiple organs are not supported by that study and should not be included without additional animal evidence.

Miltefosine provides a useful pharmacological precedent because host-mediated effects have been investigated alongside its direct antileishmanial activity. A systematic review found evidence that miltefosine can modify macrophage and T-cell responses, although the magnitude and clinical importance of these effects vary among experimental systems (Palić et al., 2019). As miltefosine is a synthetic antileishmanial drug rather than a natural product, it should be presented only as proof that direct and host-mediated mechanisms can coexist—not as evidence for natural-product immunomodulation.

Overall, niranthin and mahanine currently provide the strongest integrated evidence for host-associated parasite clearance among the compounds discussed. Diallyl sulfide has supportive animal-level evidence but was tested in combination therapy, while vanillin derivatives remain supported predominantly by infected-cell experiments. Definitive validation requires immune-pathway inhibition, cytokine neutralization or genetically modified host models demonstrating that loss of the proposed host response reduces compound efficacy. Collectively, these studies indicate that several natural products combine direct antileishmanial activity with modulation of macrophage and Th1-associated responses; however, causal evidence linking immune modulation to parasite clearance remains limited (Table 2).

**Table 2:** Natural products with experimentally reported immunomodulatory effects in leishmaniasis.

Natural product or intervention	Natural source/class	Infection model	Reported immunomodulatory effect	Effect on infection	Critical interpretation	**Ref**.
Formononetin	Natural isoflavone	*L. tropica*-infected macrophages	Increased NO release and IFN-γ and iNOS expression	Reduced macrophage infection and intracellular parasite survival	Cellular evidence only; immune-pathway dependence and animal efficacy remain untested	Mahmoudvand et al., 2023
Curcumin	Curcuma longa polyphenol	*L. major*-infected human PBMCs	Increased IFN-γ, TNF-α and iNOS transcripts	Direct parasite activity was demonstrated in a separate promastigote study	Immunomodulation is supported mainly by transcriptional measurements; intracellular killing was not linked mechanistically to these changes	Alinejad et al., 2022
Mahanine	Carbazole alkaloid from Murraya koenigii	Infected macrophages and *L. donovani*-infected mice	UCP2 suppression and increased IL-12-, iNOS-, NO- and ROS-associated responses	Reduced hepatic and splenic parasite burdens	Strong cellular and animal evidence; direct and host-mediated contributions were not quantitatively separated	Roy et al., 2017
Niranthin	Lignan from Phyllanthus amarus	*L. donovani*-infected macrophages and mice	Increased IL-12/IFN-γ-associated Th1 response and macrophage NO/ROS production	Reduced tissue burden; direct topoisomerase IB inhibition also demonstrated	Strong dual-action evidence; immune-pathway dependence was not established by cytokine neutralization	Chowdhury et al., 2012
Glycyrrhizic acid	Licorice-derived triterpenoid glycoside	*L. donovani*-infected macrophages and BALB/c mice	COX-2 suppression, increased iNOS/NO and restoration of Th1-associated responses	Reduced visceral parasite burden	Strong host-associated evidence; the contribution of direct parasite toxicity requires separation	Bhattacharjee et al., 2012
Neem leaf ethyl-acetate fraction	Defined Azadirachta indica leaf fraction	*L. donovani* promastigotes, infected macrophages and mice	Increased macrophage ROS/NO and Th1-associated transcripts with reduced Th2-associated expression	Reduced intracellular and tissue parasite burdens	Cellular and animal evidence, but the active constituent remains unidentified	Dayakar et al., 2015
Calotropis procera methanolic extract	Medicinal-plant extract	*L. major* promastigotes and infected mononuclear cells	Increased TNF-α, IFN-γ and iNOS transcription	Reduced promastigote viability; intracellular parasite clearance was not directly quantified	Preliminary promastigote activity and infection-associated transcriptional evidence using a chemically undefined extract; increased TNF-α, IFN-γ, and iNOS expression does not establish intracellular-amastigote killing or pathway dependence	Amani et al., 2024
Diallyl sulfide with meglumine antimoniate	Garlic-derived organosulfur compound	*L. major*-infected BALB/c mice	Increased IFN-γ, TNF-α, NO-associated responses and CD4+ cell infiltration	Reduced lesion size and parasite burden	Combination-treatment evidence without formal quantitative synergy analysis; the independent antiparasitic and immunomodulatory contributions of diallyl sulfide remain uncertain	Zarrinkar et al., 2024

PBMCs, peripheral blood mononuclear cells; UCP2, uncoupling protein 2; iNOS, inducible nitric oxide synthase; NO, nitric oxide; ROS, reactive oxygen species; COX-2, cyclooxygenase-2.

## 7 Host-directed therapeutic strategies: role of miRNAs and immune regulation

Host-directed therapy aims to interfere with host pathways that are exploited by intracellular pathogens for survival and persistence. This approach is relevant to leishmaniasis because *Leishmania* remodels macrophage signaling, cytokine production, antigen presentation, autophagy and antimicrobial effector functions to establish intracellular infection (Gurjar et al., 2025). Among the regulatory components involved in these responses, microRNAs (miRNAs) act as post-transcriptional regulators capable of altering multiple immune and metabolic pathways simultaneously (Acuña et al., 2020; Kumar et al., 2020b; Rashidi et al., 2021).

Profiling studies have demonstrated that *Leishmania* infection modifies host-miRNA expression in human phagocytes, experimentally infected cells and peripheral blood mononuclear cells obtained from infected animals (Geraci et al., 2015; Singh et al., 2016; Bragato et al., 2018). Functional evidence is available for selected miRNAs. For example, miR-30a-3p regulates autophagy in *L. donovani*-infected host cells, whereas parasite-induced activation of host c-Myc has been associated with broader changes in macrophage-miRNA expression (Singh et al., 2016; Nandan et al., 2021). These observations indicate that miRNA dysregulation is not merely a consequence of infection but can contribute to cellular conditions that influence parasite persistence.

Nevertheless, the therapeutic potential of miRNA modulation remains preliminary. Most available studies are based on expression profiling or experimentally infected cells, and comparatively few miRNA candidates have been validated through specific inhibition or restoration in animal models. Furthermore, because individual miRNAs can regulate numerous transcripts, therapeutic manipulation could produce unintended effects on macrophage function and systemic immunity. Accordingly, miRNAs should presently be considered mechanistically informative and potentially actionable host regulators rather than established therapeutic targets.

### 7.1 Host miRNA dysregulation and host–*Leishmania* interactions

MicroRNAs are small non-coding RNAs that regulate gene expression through transcript degradation or translational repression. During *Leishmania* infection, changes in host-miRNA expression have been detected in macrophages, CD4^+^ T cells, peripheral blood mononuclear cells and patient lesions. However, these changes vary according to parasite species, host-cell type and infection stage, and only a subset has been functionally connected to parasite survival or immune regulation (Lemaire et al., 2013; Geraci et al., 2015; Bragato et al., 2018; Rashidi et al., 2021; Lago et al., 2023).

#### 7.1.1 Species-specific miRNA dysregulation

In *L. donovani*-infected macrophages, transcriptomic studies have identified numerous differentially expressed miRNAs associated with apoptosis, phagocytosis, oxidative responses and macrophage activation (Singh et al., 2016; Tiwari et al., 2017). Nevertheless, many miRNA–target relationships reported by profiling studies were derived from computational prediction and should not be interpreted as experimentally confirmed regulation. A similar limitation applies to CD4^+^ T-cell profiling, in which infection altered miRNAs predicted to target transcription factors involved in Th1 and Th2 differentiation; although these results suggest miRNA-mediated alteration of T-cell polarization, direct validation of most predicted targets remains lacking (Kumar et al., 2020a).

Stronger functional evidence is available for selected miRNAs. *L. donovani* induces miR-30a-3p, which suppresses the autophagy-related gene *BECN1* and limits autophagic activity in infected macrophages. Inhibition of miR-30a-3p increased autophagy and reduced intracellular parasite survival, establishing a functional connection between this miRNA and infection persistence (Singh et al., 2016). The parasite also activates HIF-1α and miR-210, resulting in attenuation of NF-κB-associated inflammatory responses and increased intracellular survival (Kumar et al., 2020a). These findings support the involvement of miRNAs in parasite-mediated macrophage reprogramming rather than merely reflecting infection-associated transcriptional changes.

A distinct systemic mechanism involves hepatic miR-122. *L. donovani* GP63 targets host Dicer1, reducing miR-122 maturation and altering cholesterol metabolism in infected mice. Restoration of miR-122 or inhibition of GP63-associated signaling reduced hepatic parasite burden, providing comparatively strong mechanistic and *in vivo* evidences (Ghosh et al., 2013).

In *L. major*-infected human macrophages, miRNA profiling demonstrated time-dependent alteration of multiple miRNAs, including members of the let-7 family and miR-146a, miR-155, miR-210 and miR-132 (Lemaire et al., 2013). These findings establish infection-associated dysregulation but do not independently demonstrate that every altered miRNA promotes parasite survival. Autophagic digestion of *L. major* has also been associated with altered miR-101c, miR-129 and miR-210 expression together with changes in *BNIP3* and *CTSE*, further illustrating that the direction and functional consequence of miRNA regulation depend on the experimental system and infection stage.

Other species induce distinct regulatory patterns. In *L. amazonensis*-infected macrophages, TLR2-dependent modulation of let-7e affected inflammatory mediator production and parasite burden, providing evidence connecting a defined innate receptor–miRNA axis with infection outcome (Muxel et al., 2018). Melatonin treatment altered miR-294, miR-30e and miR-302d expression and affected *Tnf*, *Mcp1* and *Nos2* expression, although these findings represent pharmacologically modified infection rather than the unperturbed host response (Fernandes et al., 2019). Infection of human cell-line-derived macrophages with *L. infantum* and *Leishmania (Viannia)* isolates induced miR-346; inhibitor experiments supported RFX1 as a regulated target, but the reliance on differentiated cell lines limits direct clinical extrapolation (Diotallevi et al., 2018).

#### 7.1.2 Functionally investigated immunoregulatory miRNAs

miR-146a represents one of the strongest mechanistically supported examples. *L. donovani* induces miR-146a-5p through super-enhancer-associated transcription, promoting alternative macrophage polarization and conditions favorable to parasite survival (Das et al., 2021). Infected macrophages can also transfer miR-146a to neighboring cells, extending suppression of inflammatory responses beyond directly infected cells (Ganguly et al., 2022). These studies support a functional role for miR-146a in establishing a permissive macrophage environment, although its importance may vary among parasite species and tissues.

The function of miR-155 is more complex and cannot be classified as uniformly protective. In visceral infection, miR-155-deficient mice exhibited impaired immune responses and increased early *L. donovani* burden, but infection ultimately resolved, indicating that miR-155 contributes to resistance without being indispensable (Varikuti et al., 2019). In contrast, deletion of miR-155 during cutaneous *L. major* infection reduced disease severity by limiting Th2 polarization and altering dendritic-cell activity (Varikuti et al., 2021). Thus, miR-155 can support resistance in visceral leishmaniasis while promoting pathology in cutaneous infection. This species- and disease-form dependence argues against indiscriminate therapeutic delivery or inhibition of miR-155.

miR-21 has been linked to suppression of IL-12 production, but the most direct leishmaniasis evidence comes from vaccination with centrin-deficient *L. donovani*, rather than therapeutic inhibition during established virulent infection. Reduced miR-21 expression following vaccination permitted stronger IL-12-associated immunity, whereas miR-21 induction impaired this protective response (Gannavaram et al., 2019). Therefore, miR-21 is a plausible immunoregulatory target, but the manuscript should not claim that its inhibition has already reduced parasite burden during established leishmaniasis.

Clinical and veterinary studies provide additional associative evidence. Expression of miR-150 and miR-194 correlated with parasite burden in canine visceral leishmaniasis (Bragato et al., 2018), whereas altered miR-155-5p and other apoptosis-associated miRNAs were detected in lesions from patients with *L. major* or *L. tropica* infection (Masoudsinaki et al., 2025). These observations may support biomarker development, but they do not establish whether the miRNAs drive disease or reflect differences in parasite load, inflammation or lesion composition.

#### 7.1.3 Parasite-derived miRNA-like sequences

Computational analyses have predicted miRNA-like sequences in the *L. major* genome, including sequences with potential similarity to mammalian miRNAs (Sahoo et al., 2014). However, canonical RNA-interference machinery is absent from *L. major* and several other *Leishmania* species, and the predicted elements have not been adequately validated as processed, exported and functionally active small RNAs. They should therefore be described as putative miRNA-like sequences rather than parasite miRNAs. Evidence that they enter host cells, associate with host AGO2 and regulate endogenous host targets remains insufficient.

Overall, host-miRNA dysregulation is a reproducible feature of *Leishmania* infection, but the strength of evidence varies substantially. The most convincing mechanistic evidence currently concerns miR-30a-3p-mediated autophagy regulation, HIF-1α/miR-210 signaling, GP63–Dicer1–miR-122 regulation, miR-146a-mediated macrophage polarization and context-dependent functions of miR-155. Most other reported miRNAs remain supported primarily by expression profiling, target prediction or clinical correlation.

### 7.2 miRNA-mediated immune modulation as a therapeutic opportunity

The functional studies described above provide a rationale for exploring miRNAs as host-directed therapeutic targets. In principle, miRNA mimics could restore protective miRNAs suppressed during infection, whereas antisense inhibitors could block miRNAs that facilitate parasite persistence. However, the ability of individual miRNAs to regulate numerous transcripts is both an advantage and a safety concern. Broad target coverage may simultaneously influence several antiparasitic pathways, but it may also disturb unrelated immune, metabolic or proliferative processes. Consequently, reduced toxicity cannot yet be claimed as an inherent advantage of miRNA-based treatment.

#### 7.2.1 miR-155-directed interventions

A chitosan-based miR-155 polyplex has recently been evaluated in *L. major*-infected RAW264.7 macrophages. Treatment increased miR-155 expression, IL-12 production and nitric oxide release and produced a higher cell-death index in infected cultures than in untreated controls (Rahmani et al., 2025). This study demonstrates the feasibility of delivering a miRNA mimic to infected macrophages, but it was limited to an *in vitro* murine cell-line model. Moreover, the reported increase in cell death should not be interpreted as selective parasite killing because the assay reflected death of infected host cells. *In vivo* biodistribution, macrophage selectivity, systemic inflammatory effects and lesion resolution remain untested.

A different strategy combined a miR-155 inhibitor with a miR-15a mimic in *L. major* infection. Treatment promoted apoptosis in infected macrophages, and administration to BALB/c mice was associated with stabilization of lesion size and reduced parasite burden compared with untreated controls (Gholamrezaei et al., 2020). This provides proof-of-concept in an animal model, but the individual contributions of miR-155 inhibition and miR-15a replacement were not fully separated. The study also requires replication with larger cohorts, appropriate delivery controls and measurement of systemic toxicity before therapeutic conclusions can be drawn.

The opposite direction of miR-155 manipulation in these studies—replacement in one model and inhibition in another—illustrates an important biological limitation. miR-155 contributed to early resistance against visceral *L. donovani* infection but promoted susceptibility during cutaneous *L. major* infection (Varikuti et al., 2019). Therefore, miR-155 should not be described as a universally protective or universally pathogenic miRNA. Its therapeutic direction must be selected according to parasite species, disease form, immune environment and infection stage.

#### 7.2.2 Targeting host lipid metabolism

Host-lipid metabolism provides a more defined basis for miRNA-directed intervention. *L. donovani* modifies macrophage sphingolipid metabolism, and miR-330-5p was identified as a regulator of serine palmitoyltransferase long-chain base subunit 1 (SPTLC1). Infection reduced miR-330-5p expression, whereas transfection with a miR-330-5p mimic suppressed SPTLC1, increased pro-inflammatory cytokine expression and reduced intracellular parasite burden in THP-1-derived and primary human macrophages (Akand et al., 2025). Target regulation was supported by a 3′-UTR reporter assay, providing stronger mechanistic evidence than expression correlation alone. Nevertheless, efficacy has not been demonstrated in animals, and the systemic consequences of inhibiting sphingolipid synthesis require careful evaluation because this pathway is essential to membrane organization, cellular signaling and immune-cell function.

Cholesterol metabolism is regulated through a related mechanism. *L. donovani* infection increased miR-1303 and miR-874-3p, which were functionally linked to the cholesterol-biosynthetic genes *HMGCR* and *HMGCS1*, respectively. Antagonizing these miRNAs restored target-gene expression and reduced intracellular parasite burden in THP-1-derived and primary human macrophages (Tabrez et al., 2024). Reporter assays strengthened the proposed miRNA–target relationships. However, the study remained cellular, and systemic enhancement of cholesterol synthesis may produce undesirable metabolic effects. Translation would therefore require macrophage-targeted delivery rather than unrestricted administration of the inhibitors.

#### 7.2.3 miR-146a as a proposed, rather than validated, target

miR-146a-5p is another potential therapeutic target because its induction during *L. donovani* infection promotes M2-like macrophage polarization, increases arginase-associated responses and suppresses inflammatory activity (Das et al., 2021; Ganguly et al., 2022). Nevertheless, there is currently insufficient therapeutic evidence showing that pharmacological inhibition of miR-146a improves leishmaniasis *in vivo*. The previous statement that miR-146a promotes *L. major* clearance by targeting SMAD4 was based primarily on systems-level prediction rather than definitive therapeutic validation. Therefore, miR-146a should presently be described as a mechanistically supported candidate target, not an established treatment.

#### 7.2.4 Translational limitations

Collectively, these studies demonstrate that experimental manipulation of host miRNAs can alter macrophage activation, lipid metabolism and intracellular parasite survival. The strongest target-validation evidence currently concerns the miR-330-5p–SPTLC1, miR-1303–HMGCR and miR-874-3p–HMGCS1 axes, whereas the most advanced *in vivo* evidences involves combined miR-155 inhibition and miR-15a replacement. Nevertheless, no miRNA-based treatment has progressed to clinical evaluation for leishmaniasis.

Clinical translation will require selective delivery of miRNA mimics or inhibitors to infected macrophages or affected tissues, together with sufficient stability in circulation and resistance to nuclease-mediated degradation. Potential unintended immune activation and off-target regulation must also be evaluated because individual miRNAs can influence numerous transcripts and cellular pathways. Furthermore, therapeutic effects should be examined across different *Leishmania* species, clinical forms and host immune backgrounds rather than being generalized from a single experimental model. Demonstration of pharmacokinetic suitability, therapeutic efficacy and acceptable safety in disease-relevant animal models will be essential before clinical investigation can be considered. Thus, miRNA manipulation represents a promising experimental host-directed strategy, but it remains at an early preclinical stage. The experimentally investigated host-miRNA pathways, their context-dependent effects and potential strategies for therapeutic manipulation are summarized in Supplementary Figure S1.

### 7.3 Crosstalk between natural products, miRNAs and immunity

Natural products can regulate inflammatory and immune responses through transcriptional, epigenetic and metabolic mechanisms. Evidence obtained primarily from cancer and non-infectious inflammatory models indicates that phytochemicals such as curcumin, resveratrol and quercetin can alter the expression of immune-associated miRNAs, including miR-21, miR-146a and miR-155 (Milenkovic et al., 2013; Kunnumakkara et al., 2017; Kang, 2019; Malaguarnera, 2019; Wang et al., 2022). These observations provide biological plausibility for a natural product–miRNA–immunity axis. However, evidence from unrelated disease models cannot establish that the same miRNA-dependent mechanisms contribute to parasite clearance during leishmaniasis.

Melatonin provides one of the few direct examples in an experimental *Leishmania* infection. In *L. amazonensis*-infected macrophages, melatonin altered miR-294, miR-30e and miR-302d expression, accompanied by changes in *Tnf*, *Mcp1* and *Nos2* expression and intracellular parasite burden (Fernandes et al., 2019). This study connects exposure to a naturally occurring compound with miRNA regulation and macrophage responses during infection. Nevertheless, miRNA mimics, inhibitors or rescue experiments were not used to determine whether the changes in these miRNAs were required for the antileishmanial effect. The findings therefore demonstrate an association, but not a causal natural compound–miRNA–parasite-clearance pathway.

A similar distinction is required for phytochemicals already discussed in this review. Curcumin increases IFN-γ, TNF-α and iNOS expression in *L. major*-infected peripheral blood mononuclear cells (Alinejad et al., 2022), whereas *Calotropis procera* extract increases ROS and Th1-associated inflammatory transcripts in infected cells (Amani et al., 2024). Curcumin and other polyphenols regulate miRNA expression in cancer and inflammatory models (Milenkovic et al., 2013; Kunnumakkara et al., 2017; Kang, 2019), but no study has demonstrated that their antileishmanial immunomodulatory activity depends on a defined miRNA. Likewise, quercetin regulates non-coding RNA networks in cancer models (Wang et al., 2022), but its direct antileishmanial effects cannot presently be attributed to miRNA modulation.

### 7.4 Critical evidence assessment and research gaps

Current evidence supports natural-product immunomodulation and host-miRNA regulation as two biologically relevant but largely parallel research areas. The central unresolved question is whether natural products reduce *Leishmania* burden by regulating specific host miRNAs or whether the observed miRNA changes are secondary consequences of reduced infection, oxidative stress or immune activation. Except for limited evidence from studies such as the melatonin investigation, natural product-induced miRNA changes have rarely been measured directly in *Leishmania*-infected cells. Moreover, candidate miRNA targets are frequently inferred computationally rather than confirmed through reporter assays, protein-level measurements or pathway-interruption experiments.

Few studies have used miRNA mimics, inhibitors or genetic models to establish whether a proposed miRNA is required for the antileishmanial activity of a natural compound. Direct parasite toxicity is also rarely distinguished from host miRNA-mediated activity, making it difficult to determine whether miRNA dysregulation represents a mechanism of action or a secondary response to reduced parasite burden. The available evidence is concentrated largely in cultured macrophages, with limited validation in animal models and no clinical confirmation. Species- and disease-form-specific effects remain insufficiently addressed, while the pharmacokinetics, tissue distribution and macrophage-specific delivery of miRNA-modulating compounds are largely unknown.

A rigorous demonstration of natural product–miRNA crosstalk would require showing that a compound reproducibly alters a defined miRNA in infected macrophages, changes a directly validated molecular target, modifies a relevant immune or metabolic pathway and reduces intracellular amastigote burden. Most importantly, the antileishmanial effect should be lost, attenuated or reversed following miRNA inhibition, mimic treatment or genetic manipulation. Without such pathway-interruption experiments, miRNA regulation should be described as an associated response rather than an established mechanism of action.

Overall, the natural product–miRNA interface represents a promising but insufficiently investigated area of host-directed antileishmanial therapy. Its principal value in the present review is therefore not as an established therapeutic mechanism, but as a clearly defined research opportunity integrating natural-product pharmacology, macrophage biology and miRNA-mediated immune regulation.

## 8 Combination and adjunct therapeutic approaches

Combination therapy provides a strategy for improving antileishmanial efficacy by pairing agents with complementary mechanisms of action. Potential advantages include dose reduction, delayed development of resistance and activity against parasites with reduced susceptibility to individual drugs. However, these benefits cannot be presumed because combinations may be additive, indifferent or antagonistic and may also produce cumulative toxicity. Each regimen must therefore be evaluated through quantitative interaction analyses and direct comparison with its individual components.

### 8.1 Conventional antileishmanial drug combinations

Clinical evidence supports selected combinations of established antileishmanial drugs. Liposomal amphotericin B combined with a short course of miltefosine produced a high cure rate in Indian visceral leishmaniasis and performed favorably compared with miltefosine monotherapy (Goswami et al., 2020). Nevertheless, the study evaluated a particular liposomal amphotericin B formulation, and its findings may not be directly transferable to other formulations, geographical regions, parasite populations or immunocompromised patients.

Miltefosine combined with paromomycin has also been investigated as a resistance-limiting strategy. In an experimental *L. infantum* model, combined exposure delayed the emergence of resistance compared with either agent alone (Hendrickx et al., 2017). This supports the principle that compounds with different mechanisms can increase the evolutionary barrier to resistance. However, delayed resistance under laboratory selection does not establish that the same effect will occur during clinical treatment, where drug exposure, host immunity and adherence introduce additional variables.

Combination therapy should therefore be described as regimen-specific rather than universally superior. Potential benefits must be balanced against cumulative toxicity, pharmacokinetic incompatibility, increased treatment cost and the difficulty of maintaining adherence when the component drugs require different routes or schedules.

### 8.2 Natural product–drug combinations

Natural products may serve as adjuncts by contributing direct antiparasitic activity, modifying host responses or increasing parasite susceptibility to conventional drugs. Among the better-investigated examples, apigenin has been evaluated in combination with miltefosine against experimental cutaneous leishmaniasis. Fractional inhibitory concentration analysis produced a ΣFIC value of 1.61, indicating an additive rather than synergistic interaction *in vitro*. In *L. amazonensis*-infected mice, treatment with reduced doses of both agents decreased lesion size and parasite burden. These findings provide animal-level proof of concept for a combined effect, but they do not establish synergism, and clinical efficacy, pharmacokinetic interactions and long-term recurrence were not evaluated (Emiliano and Almeida-Amaral, 2018).

Diallyl sulfide, an organosulfur constituent of garlic, has been studied in combination with meglumine antimoniate against *L. major*. The combination reduced lesion size and parasite burden in an animal model and was accompanied by increased IFN-γ, TNF-α, nitric oxide and CD4^+^-cell infiltration (Zarrinkar et al., 2024). This suggests combined antiparasitic and host-associated effects. However, the immune changes do not prove that immunomodulation was necessary for treatment efficacy, and the independent contribution of diallyl sulfide requires further separation from that of meglumine antimoniate. Because no fractional inhibitory concentration, isobologram, or combination-index analysis was reported, the findings should be described as a combined or enhanced effect rather than synergism.

Gallic acid has recently been evaluated with paromomycin against intracellular *L. (Viannia) guyanensis*. The study assessed antiparasitic activity, macrophage cytotoxicity and human immune-cell responses (Hanada et al., 2026). However, gallic acid alone demonstrated greater activity against intracellular amastigotes than the reported combination, and formal synergy was not established. This combination should therefore be presented as an early compatibility study rather than evidence of a superior regimen.

These findings emphasize that improved activity relative to an untreated control is insufficient to demonstrate synergy. A synergistic interaction must be established using quantitative methods such as fractional inhibitory concentration indices, isobologram analysis or combination-index modeling and should subsequently be confirmed in intracellular and animal models.

### 8.3 Nanoparticle- and formulation-assisted combinations

Nano-formulations, liposomes and polymeric delivery systems may increase the solubility, stability and macrophage uptake of natural compounds or conventional drugs (Nafari et al., 2020). Macrophage-directed delivery is particularly relevant because intracellular amastigotes reside within parasitophorous vacuoles, where adequate drug exposure can be difficult to achieve.

Nevertheless, nanoparticle incorporation should not itself be interpreted as evidence of improved treatment. Comparative studies must demonstrate that the formulation increases intracellular drug exposure or therapeutic efficacy relative to the free compound without introducing additional toxicity. Particle composition, size, surface charge, drug-loading efficiency, release kinetics, biodistribution and clearance can substantially influence biological performance. Most natural product–nanoparticle combinations remain supported by *in vitro* or small-animal studies, with limited information on reproducibility, large-scale manufacturing or long-term safety.

### 8.4 Critical evidence assessment and research priorities

Natural product–drug combinations remain considerably less developed than combinations involving established antileishmanial agents. Apigenin combined with miltefosine and diallyl sulfide combined with meglumine antimoniate currently provide relevant animal-level evidence, whereas the gallic acid–paromomycin combination is supported primarily by *in vitro* findings (Emiliano and Almeida-Amaral, 2018; Zarrinkar et al., 2024; Hanada et al., 2026). None of these natural product–drug combinations has yet been validated in a controlled clinical trial. Their therapeutic value therefore remains provisional.

Future studies should prioritize activity against intracellular amastigotes rather than relying predominantly on promastigote assays. Each combination should be compared with its individual components at equivalent doses, followed by formal assessment of synergistic, additive or antagonistic interactions using fractional inhibitory concentration indices, isobologram analysis or combination-index modeling. An improved outcome relative to an untreated control does not establish synergy unless the combination also demonstrates an advantage over appropriately dosed individual treatments.

Combinations showing reproducible intracellular activity should subsequently undergo pharmacokinetic and tissue-distribution analyses to determine whether both components reach effective concentrations at infected sites. Evaluation should include acute and repeated-dose toxicity, potential pharmacokinetic incompatibility and immune-mediated adverse effects. Efficacy must then be confirmed across relevant *Leishmania* species, drug-resistant isolates and disease-appropriate animal models. Claims that a combination reduces toxicity, shortens treatment or delays resistance should be made only when these outcomes have been measured directly.

Standardization is particularly important for combinations containing botanical extracts because their chemical composition may vary with plant species, geographical origin, cultivation, harvesting, extraction and storage conditions. Mechanistic and translational studies should therefore preferentially use purified compounds or chemically standardized fractions with defined marker constituents and demonstrated batch-to-batch consistency.

Overall, conventional drug combinations retain the strongest clinical foundation, whereas natural product–drug and formulation-assisted combinations remain predominantly preclinical. Translation will require evidence that a proposed combination provides a reproducible and clinically meaningful advantage over its individual components without introducing unacceptable toxicity, formulation complexity or cost.

## 9 Challenges, knowledge gaps and future directions

Despite the large number of natural products reported to possess antileishmanial activity, most candidates remain at an early preclinical stage. The principal translational barriers are weak experimental models, incomplete chemical standardization, uncertain molecular targets, poor pharmacokinetics and limited therapeutic validation. Host-directed and miRNA-based approaches introduce additional challenges related to species-specific immune responses, off-target effects and selective delivery.

### 9.1 Limitations of the available evidence

A substantial proportion of natural-product studies rely on promastigote assays. Promastigotes are useful for preliminary screening but differ biologically and metabolically from intracellular amastigotes, which represent the clinically relevant stage in mammalian infection. Compounds active only against promastigotes should therefore be considered screening hits rather than validated antileishmanial candidates.

Even when intracellular activity is reported, studies frequently use a single parasite strain and immortalized macrophage cell line. This limits generalization across *Leishmania* species, clinical forms and drug-resistant isolates. Species-specific differences in parasite metabolism, host-cell preference and immune evasion mean that activity against one species cannot be assumed to extend to others. Future studies should prioritize intracellular amastigotes, primary macrophages, clinically relevant isolates and appropriate animal models.

Another recurring limitation is the absence of standardized evidence thresholds. Reductions in parasite number, increases in ROS or NO and changes in cytokine expression are often interpreted as proof of mechanism. However, these findings establish association unless the proposed target or pathway is disrupted through genetic, biochemical or pharmacological experiments. Target engagement, pathway dependence and rescue experiments should therefore accompany phenotypic efficacy studies.

### 9.2 Chemical standardization and pharmacokinetic barriers

Crude extracts can contain numerous constituents whose concentrations vary according to plant genotype, cultivation, harvesting, extraction and storage. This variability limits reproducibility and makes it difficult to identify the active compound, establish structure–activity relationships or define a clinically reproducible dose (Liu, 2008; Atanasov et al., 2015). Extract-based studies should therefore report botanical authentication, extraction procedures, chemical fingerprints, marker-compound concentrations and batch-to-batch consistency.

Purification of individual constituents can improve standardization, but it may also remove pharmacologically relevant interactions among extract components. Comparisons among the crude extract, standardized fraction and purified active compound are needed to determine whether activity depends on a single constituent or a reproducible combination.

Many phytochemicals also exhibit poor aqueous solubility, rapid metabolism and limited tissue exposure. Curcumin is a representative example for which extensive biological activity has been reported despite low systemic bioavailability (Siviero et al., 2015). Similar concerns apply to other polyphenols, including quercetin, resveratrol and EGCG (Cione et al., 2019). High activity in cell culture is unlikely to translate if the required concentration cannot be achieved in infected skin, liver, spleen or bone marrow.

Nanocarriers and liposomal formulations may improve solubility, stability and macrophage uptake, but they do not automatically resolve these limitations. Nanoparticle size, charge, composition, release kinetics and biodistribution can alter both efficacy and toxicity. Scalability, manufacturing reproducibility, long-term safety and regulatory requirements remain substantial barriers (Nafari et al., 2020; Vaiserman et al., 2020).

### 9.3 Mechanistic and host-directed therapeutic gaps

Many natural products are described as multitarget agents. Although multitarget activity may be therapeutically useful, it can also reflect incomplete target identification. Claims that multitarget activity will prevent resistance remain speculative unless candidates are tested under prolonged drug pressure and against resistant clinical isolates. *Leishmania* can adapt through altered gene dosage, chromosome copy-number variation, transporter regulation and metabolic remodeling, making resistance assessment an essential component of development (Singh et al., 2019; Majoor et al., 2025).

Host-directed therapy offers an alternative approach by targeting macrophage pathways required for parasite survival or clearance (Singh et al., 2019). However, host pathways frequently perform essential physiological functions. Manipulating autophagy, arginine metabolism, macrophage polarization or inflammatory cytokines may improve parasite control but could also cause tissue damage, systemic inflammation or immunosuppression. Therapeutic windows must therefore be defined experimentally rather than inferred from parasite reduction alone.

miRNA-based interventions face similar concerns. Individual miRNAs regulate numerous transcripts, and their functions can vary according to parasite species, tissue and infection stage. The contrasting roles of miR-155 in visceral and cutaneous models demonstrate that the same intervention may produce different outcomes in different forms of leishmaniasis. In addition, stable and macrophage-selective delivery of miRNA mimics or inhibitors remains unresolved, while systemic delivery may affect unrelated tissues and immune pathways (Rupaimoole and Slack, 2017).

The natural product–miRNA relationship represents a particularly important research gap. Current evidence does not establish that the antileishmanial activity of commonly studied phytochemicals depends on specific miRNAs. Future studies must combine compound treatment with miRNA inhibition, replacement or genetic manipulation to distinguish causal regulation from secondary expression changes.

### 9.4 Priorities for future research

Future research should follow a rational translational pathway beginning with botanical authentication, chemical characterization and batch standardization of extracts or purified compounds. Candidate products should then be evaluated against intracellular amastigotes in primary host cells and across multiple *Leishmania* species, clinical isolates and drug-resistant strains. Compounds showing reproducible activity should undergo direct target and pathway validation using biochemical assays, genetic manipulation, target-engagement measurements and rescue experiments rather than relying solely on molecular docking or changes in gene expression. Their activity should also be compared with established antileishmanial drugs using clinically relevant concentrations and appropriate selectivity indices.

The strongest candidates should subsequently be examined through pharmacokinetic, pharmacodynamic and tissue-distribution studies to determine whether effective concentrations can be achieved in infected skin, liver, spleen or bone marrow. Acute and repeated-dose toxicity, immune-mediated adverse effects and interactions with existing antileishmanial treatments should be assessed before efficacy is evaluated in disease-relevant animal models. Candidates that retain efficacy and acceptable safety should then progress to standardized formulation, manufacturing assessment and, ultimately, controlled clinical evaluation.

Multi-omics and computational approaches may assist target identification, compound prioritization and biomarker development. However, molecular docking, network analysis and artificial intelligence should be regarded as hypothesis-generating tools rather than experimental evidence of target binding or therapeutic efficacy. Computational predictions must therefore be followed by biochemical, cellular and *in vivo* validation.

Combination therapies should similarly undergo formal interaction analysis. Claims of synergy require comparison with each individual component and quantitative determination of synergistic, additive or antagonistic effects. Reduced toxicity, shortened treatment duration or prevention of resistance should be claimed only when these outcomes have been measured directly.

Finally, clinical development should consider affordability, formulation stability, route of administration and feasibility in endemic regions. A compound with strong experimental activity may have limited practical value if it requires complex manufacturing, continuous refrigeration or prolonged parenteral administration. Translation will therefore require coordination among parasitology, immunology, medicinal chemistry, pharmacology, formulation science and clinical research.

## 10 Conclusion

Natural products represent a chemically diverse source of antileishmanial candidates capable of acting through direct parasite toxicity, inhibition of parasite-specific metabolic pathways and modulation of host immune responses. Experimental evidence supports several mechanisms, including disruption of redox homeostasis and mitochondrial function, inhibition of arginase and polyamine metabolism, interference with parasite replication and enhancement of macrophage-mediated parasite clearance. However, the strength of evidence varies substantially among compounds, and many proposed mechanisms remain based on promastigote assays, molecular docking or expression-level associations rather than direct target validation.

Host-directed approaches, particularly those involving macrophage activation and miRNA regulation, broaden the therapeutic potential of natural products but remain at an early preclinical stage. Although selected miRNAs have been functionally linked to parasite persistence or clearance, their effects are often dependent on parasite species, disease form and host-cell context. Evidence that natural products exert antileishmanial activity through specific miRNA-dependent mechanisms is currently limited and represents an important research gap.

Future progress will depend on prioritizing chemically standardized candidates with reproducible activity against intracellular amastigotes, validated molecular targets, acceptable selectivity and achievable exposure in infected tissues. Pharmacokinetic, toxicological and *in vivo* studies should precede clinical evaluation, while proposed combination therapies must demonstrate a measurable advantage over their individual components. Overall, natural products and host-directed interventions offer promising complementary strategies for leishmaniasis, but their clinical translation will require a shift from descriptive screening toward mechanism-based, standardized and clinically relevant investigation.

## Supplementary material

Supplementary Figure S1 Host miRNA regulation and potential host-directed interventions during Leishmania infection *Leishmania donovani*–induced miR-30a-3p suppresses BECN1-associated autophagy and supports intracellular parasite survival (Singh et al., 2016). Parasite GP63-mediated reduction of hepatic Dicer1 and miR-122 remodels cholesterol metabolism and facilitates liver infection (Ghosh et al., 2013). Restoration of miR-330-5p suppresses SPTLC1 and reduces intracellular parasite burden (Akand et al., 2025), whereas inhibition of infection-induced miR-1303 and miR-874-3p restores HMGCR and HMGCS1 expression and limits parasite survival (Tabrez et al., 2024). The function of miR-155 is context-dependent, contributing to early protection during visceral *L. donovani* infection but promoting susceptibility during cutaneous *L. major* infection (Varikuti et al., 2019, 2021). miRNA mimics, inhibitors and macrophage-targeted delivery systems represent potential host-directed interventions. The dashed natural-product pathway denotes an unresolved relationship: melatonin alters host-miRNA expression during *L. amazonensis* infection, but causal miRNA dependence has not been demonstrated (Fernandes et al., 2019). Arrows indicate activation or increased expression; T-bars indicate inhibition.
